# A Phenotyping Tool for Seven Cytochrome P450 Enzymes and Two Transporters: Application to Examine the Effects of Clopidogrel and Gemfibrozil

**DOI:** 10.1002/cpt.3610

**Published:** 2025-02-21

**Authors:** Laura Aurinsalo, Outi Lapatto‐Reiniluoto, Mika Kurkela, Mikko Neuvonen, Johanna I. Kiiski, Mikko Niemi, Aleksi Tornio, Janne T. Backman

**Affiliations:** ^1^ Department of Clinical Pharmacology University of Helsinki Helsinki Finland; ^2^ Individualized Drug Therapy Research Program, Faculty of Medicine University of Helsinki Helsinki Finland; ^3^ Department of Clinical Pharmacology, HUS Diagnostic Center Helsinki University Hospital Helsinki Finland; ^4^ HUS Pharmacy Helsinki University Hospital Helsinki Finland; ^5^ Integrative Physiology and Pharmacology, Institute of Biomedicine University of Turku Turku Finland; ^6^ Unit of Clinical Pharmacology Turku University Hospital Turku Finland

## Abstract

Clinical cocktails for cytochrome P450 (CYP) phenotyping lack a marker for CYP2C8. We aimed to combine the CYP2C8 index drug repaglinide with the Geneva cocktail (caffeine/CYP1A2, bupropion/CYP2B6, flurbiprofen/CYP2C9, omeprazole/CYP2C19, dextromethorphan/CYP2D6, and midazolam/CYP3A4). We also included endogenous organic anion transporting polypeptide (OATP) 1B1 and 1B3 biomarkers glycochenodeoxycholate 3‐O‐glucuronide and glycochenodeoxycholate 3‐sulfate, and investigated the CYP2C8 inhibition selectivity of clopidogrel and gemfibrozil with the full cocktail. In a five‐phase randomized cross‐over study, the following drugs were administered to 16 healthy volunteers: (i) repaglinide, (ii) the Geneva cocktail, (iii) repaglinide with the Geneva cocktail (full cocktail), (iv) clopidogrel followed by the full cocktail, and (v) gemfibrozil followed by the full cocktail. The Geneva cocktail increased repaglinide AUC_0‐23h_ 1.22‐fold (90% confidence interval 1.04–1.44, *P* = 0.033). The full cocktail accurately captured known inhibitory effects of clopidogrel on CYP2B6, CYP2C8, and CYP2C19 and that of gemfibrozil on CYP2C8. Gemfibrozil decreased the paraxanthine/caffeine AUC_0‐12h_ ratio by 23% (14–31%, *P* < 0.01) and increased caffeine AUC_0‐12h_ 1.20‐fold (1.03–1.40, *P* = 0.036). Gemfibrozil increased the metabolite‐to‐index drug AUC_0‐23h_ ratios of flurbiprofen, omeprazole, dextromethorphan, and midazolam 1.59‐fold (1.32–1.92), 1.47‐fold (1.34–1.61), 1.79‐fold (1.23–2.59), and 2.1‐fold (1.9–2.4), respectively, without affecting the index drug AUCs (*P* < 0.01). Gemfibrozil increased the AUC_0‐4h_ of glycochenodeoxycholate 3‐O‐glucuronide 1.33‐fold (1.07–1.65, *P* = 0.027). In conclusion, the combination of repaglinide, the Geneva cocktail and endogenous biomarkers for OATP1B1 and OATP1B3 yields a nine‐in‐one phenotyping tool. Apart from strong CYP2C8 inhibition, gemfibrozil weakly inhibits CYP1A2 and OATP1B1 and appears to impair the elimination of the metabolites of several CYP index drugs.

**Study Highlights**

**WHAT IS THE CURRENT KNOWLEDGE ON THE TOPIC?**

Clinical cytochrome P450 (CYP) phenotyping cocktails lack an index of CYP2C8, an important drug‐metabolizing enzyme. Although clopidogrel and gemfibrozil are index inhibitors of CYP2C8, their selectivity has not been determined clinically. Gemfibrozil has been suspected to inhibit also organic anion transporting polypeptide (OATP) 1B1.

**WHAT QUESTION DID THIS STUDY ADDRESS?**

We validated a cocktail of seven major CYP enzymes and two transporter activities by combining the CYP2C8 index drug repaglinide with the Geneva cocktail and endogenous biomarkers for OATP1B1 and OATP1B3. The effects of clopidogrel and gemfibrozil on this full cocktail were investigated to determine their selectivity as CYP2C8 index inhibitors.

**WHAT DOES THIS STUDY ADD TO OUR KNOWLEDGE?**

Repaglinide did not have relevant interactions with the Geneva cocktail. Established CYP inhibitory effects of clopidogrel and gemfibrozil were captured accurately with the full cocktail. The results documented weak inhibition of CYP1A2 and OATP1B1 by gemfibrozil. Unexpectedly, gemfibrozil increased 4′‐hydroxyflurbiprofen, 5′‐hydroxyomeprazole, dextrorphan, and 1′‐hydroxymidazolam concentrations without affecting parent drug variables. Gemfibrozil likely interferes with the elimination of these index metabolites and thus affects drug elimination pathways more than previously considered.

**HOW MIGHT THIS CHANGE CLINICAL PHARMACOLOGY OR TRANSLATIONAL SCIENCE?**

Repaglinide combined with the Geneva cocktail and endogenous biomarkers for OATP1B1 and OATP1B3 yields a phenotyping tool for simultaneous activity measurements of seven CYP enzymes and two transporters. Gemfibrozil is a more suitable CYP2C8 index inhibitor than clopidogrel as gemfibrozil is stronger and has no moderate/strong inhibitory effects on other CYPs. Yet, weak inhibition of CYP1A2 and OATP1B1 and apparent effects on uncharacterized “phase II” elimination mechanisms by gemfibrozil warrant caution in the interpretation of studies with gemfibrozil as a CYP2C8 index inhibitor.

Individual drug response is determined by genetics and epigenetics, diseases, and general physiological state, as well as various environmental factors, such as drug–drug interactions, diet, and other environmental exposures. To increase understanding of such interindividual variability and improve individualization of drug treatments, there is a need for methods to phenotype drug metabolism. Ultimately, such methods could be used to maximize the effectiveness of drug treatments and reduce adverse effects and costs.

Standalone drug–drug interaction studies utilizing sensitive index drugs are the gold‐standard for investigating a possible inhibitor or inducer of cytochrome P450 (CYP) enzymes.^1^ For profiling of drug metabolism, combinations of multiple index drugs for different CYP enzymes, called CYP cocktails, have proven useful. As activities of several CYP enzymes can be measured in one clinical trial, a more comprehensive view on drug metabolism is achieved, while efforts, costs, and invasiveness to study participants are reduced compared with studying each index drug separately. In addition to studying drug–drug interactions, index drug cocktails can be used to phenotype drug metabolism in clinical trials and patient care.^2^

Selective and sensitive clinical index drugs have been validated for all major CYP enzymes.^1^ However, it has been challenging to develop optimized index drug cocktails, for example, due to mutual interactions between index drugs, adverse effects caused by the cocktail or unfeasible sampling needed.^3^, ^4^, ^5^, ^6^, ^7^, ^8^, ^9^ In addition, CYP2C8 is not included in any of the published clinical phenotyping cocktails, such as the well‐validated Geneva cocktail, which lacks significant safety issues and includes index drugs for CYP1A2, CYP2B6, CYP2C9, CYP2C19, CYP2D6, and CYP3A.^5^, ^6^, ^10^ CYP2C8 is of importance in the metabolism of multiple clinically used drugs such as daprodustat, desloratadine, enzalutamide, hydroxychloroquine, imatinib, montelukast, paclitaxel, and pioglitazone.^11^, ^12^ Overall, CYP2C8 is the most important CYP enzyme missing from clinical cocktails.

Our aim was to add the well‐established CYP2C8 index drug repaglinide to the Geneva cocktail and to test if its addition causes any mutual interaction with the index drugs (**Table** **1**).^5^, ^6^, ^10^, ^12^ Repaglinide was chosen, because it has been extensively used, and because its short half‐life enables a short blood sampling period. We tested the effect of the moderate and strong CYP2C8 inhibitors clopidogrel and gemfibrozil^13^, ^14^ on the full cocktail to characterize the selectivity of their CYP2C8 inhibitory effect and to further test the performance of repaglinide in this CYP cocktail. As both gemfibrozil and clopidogrel have been suggested to inhibit organic anion transporting polypeptides (OATP),^15^, ^16^, ^17^ endogenous biomarkers glycochenodeoxycholate 3‐O‐glucuronide (GCDCA‐3G) for OATP1B1 and glycochenodeoxycholate 3‐sulfate (GCDCA‐3S) for OATP1B3 were measured.^18^, ^19^

**Table 1:** Pretreatment and index drugs of a five‐phase randomized open‐label controlled crossover clinical trial with 16 healthy volunteers

Study phase	Pretreatment	Index drugs
I	Placebo	Repaglinide 0.05 mg
II	Placebo	Geneva cocktail^a^
III	Placebo	Geneva cocktail^a^ + repaglinide 0.05 mg
IV	Clopidogrel 300 mg	Geneva cocktail^a^ + repaglinide 0.05 mg
V	Gemfibrozil 600 mg b.i.d for 3 days	Geneva cocktail^a^ + repaglinide 0.05 mg

Randomization was carried out using an electronic system by the Helsinki and Uusimaa Hospital District Pharmacy. All drugs were given orally as single doses if not otherwise indicated. Pretreatment drugs were administered at 8.00 am on study days (in phase V third day of gemfibrozil pretreatment) if not otherwise indicated and after that index drugs were given at 9.00 am.

^a^ Geneva cocktail contains the following CYP enzyme index drugs: 50 mg caffeine (CYP1A2), 20 mg bupropion (CYP2B6), 10 mg flurbiprofen (CYP2C9), 10 mg omeprazole (CYP2C19), 10 mg dextromethorphan (CYP2D6) and 1 mg midazolam (CYP3A4).

## METHODS

### Study design

This open‐label cross‐over clinical trial included five study phases that each participant underwent in a randomized order (**Table** **1**). There was at least 2‐week washout period between different study days. The pretreatment included placebo, clopidogrel (Plavix 300 mg, Sanofi‐Aventis, Paris, France), or gemfibrozil (Gevilon 600 mg, Pfizer Pharma GmbH, Berlin, Germany) depending on the study phase (**Table** **1**). As for the index drugs, the Helsinki and Uusimaa Hospital District Pharmacy manufactured 0.05 mg repaglinide capsules, 20 mg bupropion capsules, and 10 mg flurbiprofen capsules from the following commercially available preparations: Repaglinide Krka 0.5 mg tablet (KRKA, Novo Mesto, Slovenia), Bupropion Hydrochloride tablet 100 mg (Heritage Pharmaceuticals Inc., Eatontown, NJ), and Cebutid 50 mg tablet (Almirall SA, Barcelona, Spain), respectively, with the addition of microcrystalline cellulose for repaglinide and bupropion capsules and lactose for flurbiprofen capsules. The other index drugs were administered as commercially available preparations: caffeine (exact half of Caffeine 100 mg tablet, University Pharmacy, Helsinki, Finland), omeprazole (gastro‐resistant Losec Mups 10 mg tablet, Astra Zeneca, Cambridge, UK), dextromethorphan (5.0 mL of Rometor Ratiopharm 2 mg/mL oral solution, Ratiopharm, Ulm, Germany), and midazolam (1.00 mL of Midazolam Accord 1 mg/mL solution, Accord Healthcare, Utrecht, the Netherlands).

On study days, venous blood samples from all participants were drawn from forearm vein cannulas before the administration of the pretreatment drugs and 5 minutes before and 0.5, 1, 1.5, 2, 3, 4, 6, 8, 12, and 23 hours after the administration of the index drugs for determination of index drug and metabolite concentrations. Additional venous blood samples for biomarker analyses were drawn before the administration of the pretreatment drugs and 5 minutes before and 3 hours after the administration of the index drugs. Standardized breakfast was served 1 hour, lunch 3 hours, and snacks 7 and 9 hours after the administration of index drugs. Blood glucose levels were monitored with CareSens Dual point‐of‐care blood glucose meter (i‐SENS Inc., Seoul, South Korea) at each venous blood sampling time point up to 12 hours after the administration of index drugs.

The use of grapefruit juice was prohibited from 1 week before and throughout the study. The participants were required to abstain from alcohol the day before the study day, on study day and the day after the study day during each study phase. The consumption of caffeine containing beverages was prohibited from 10 am on the day before the study day until 21.30 pm on the study day. Participants were required to fast overnight before the study days.

### Study participants

Sixteen healthy volunteers were enrolled into this clinical trial after giving written informed consent. The participants did not take any continuous systemic medications, including hormonal contraception, were non‐smokers and had no clinically significant abnormalities in clinical examination or laboratory tests (hemoglobin, erythrocyte, leukocyte, and thrombocyte counts, liver function tests, creatinine, potassium, and sodium levels and blood glucose value). All female participants had a negative pregnancy test before each of the study phases. None of the participants had a higher blood pressure level than 145/90 mmHg or a history of abnormal bleeding.

### Analytical methods

Prior to the analysis, the analytes were extracted from the plasma samples with simple protein precipitation. Plasma samples (50 μL) were mixed with acetonitrile solution (200 μL) containing internal standards. The samples were centrifugated and an aliquot of the supernatant was mixed with an equal volume of water before LC–MS analyses.

The analytical instrumentation, for measurement of the index drugs and their corresponding metabolites for CYP1A2, CYP2B6, CYP2C9, CYP2C19, CYP2D6, and CYP3A and the pretreatment drug clopidogrel (**Table** **S1**), consisted of ExionLC liquid chromatography system coupled to the Sciex 6500 Qtrap ‐tandem mass spectrometry (AB Sciex, Toronto, ON, Canada). Analytes were separated on Atlantis T3 column (3 μm particle size, 2.1 × 100 mm, Waters, Milford, MA, USA), using a gradient elution of mobile phase consisting of 0.1% formic acid (A) and methanol (B). The flow rate was 0.3 mL/min, and the gradient program for the mobile phase B was from 20% to 90% over 7 minutes, wash at 90% 7–9.5 minutes, followed by balancing at 20% before next injection. The pretreatment drug gemfibrozil and metabolites gemfibrozil 1‐O‐β‐glucuronide, clopidogrel carboxylic acid, and clopidogrel acyl‐β‐D‐glucuronide were determined using a liquid chromatography system (Nexera X2, Shimadzu, Kyoto, Japan) coupled to an API 3000 tandem mass spectrometer (AB Sciex, Toronto, ON, Canada) (**Table** **S1**). The column and mobile phases were the same as above, and the gradient program for the mobile phase B was from 40% to 90% over 5 minutes, wash at 90% 5–7.3 minutes, followed by balancing at 40% before next injection. The quality control samples for each analyte at relevant concentrations were quantified with each batch of plasma samples. The precisions (coefficient of variation %) were below 11% and the accuracies ranged from 85% to 115%.

Plasma repaglinide, OATP1B1 biomarker GCDCA‐3G, OATP1B3 biomarker GCDCA‐3S, and CYP2D6 biomarker solanidine were analyzed using Sciex 5500 Qtrap and Sciex 6500 Qtrap LC–MS systems (AB Sciex, Toronto, ON, Canada) as previously described.^20^, ^21^, ^22^ The limit of quantification for repaglinide was 0.01 ng/mL. The between‐day precisions (CV%) for quality controls (0.1 and 2.0 ng/mL) were below 10% and accuracies were within ±10%. For GCDCA‐3G and GCDCA‐3S, the limit of quantification was 0.5 ng/mL and the between‐day precisions and accuracies were below 5% and ± 10% at relevant concentrations (10 and 200 ng/mL). For solanidine, the limit of quantification was 0.025 ng/mL and the between‐day precisions (CV%) and mean accuracies (%) were below 9% and within 5% at relevant concentrations (0.1, 0.5, and 10 ng/mL, *n* = 6).

### Genotyping

An accredited clinical pharmacogenetic panel test was used.^23^ Further details are found in **Supplementary methods**.

### Pharmacokinetics

Pharmacokinetic values, including peak plasma concentration (*C*_max_), time to *C*_max_ (*T*_max_), elimination half‐life (*t*_1/2_), and area under the concentration–time curve up to 4, 12, and 23 hours and infinity (AUC_0‐4h_, AUC_0‐12h_, AUC_0‐23h_, and AUC_0‐∞_ respectively) were calculated with Phoenix WinNonlin, Version 8.3 (Certara, Princeton, NJ) using standard non‐compartmental methods for the index drugs and their metabolites. Additionally, metabolic ratios (*C*_metabolite_/*C*_index drug_) at 2‐hour and 4‐hour timepoints and respective metabolite/index drug AUC_0‐4h_, AUC_0‐12h_, AUC_0‐23h_, and AUC_0‐∞_ ratios were calculated. As the estimated part of the AUC_0‐∞_ exceeded 10% for some index drugs and metabolites, AUC_0‐23h_ was used as the main pharmacokinetic metric for the index drugs and metabolites. The only exception was the use of AUC_0‐12h_ for caffeine and paraxanthine (caffeine consumption was allowed after collection of 12‐hour sample) (**Table** **S2**). For clopidogrel, gemfibrozil and their metabolites *C*_max_, *T*_max_, *t*_1/2_ and AUC_0‐9h_ or AUC_0‐24h_ values were calculated with Phoenix WinNonlin, Version 8.3 (Certara, Princeton, NJ) using standard non‐compartmental methods. As for biomarkers, AUC_0‐3h_ values in phases I–III and AUC_0‐4h_ values in phases III–V for GCDCA‐3G and GCDCA‐3S and AUC_0‐4h_ values in phases III and V for M430, M444, and solanidine were calculated with trapezoidal rule in Excel (Microsoft, Redmond, WA). 0‐hour metabolic ratio and AUC_0‐4h_ ratios were calculated for M430/solanidine and M444/solanidine.

### Statistical analyses

The sample size of 16 participants was estimated to be sufficient to detect a 25% difference in the AUCs and AUC ratios of repaglinide and the Geneva cocktail index drugs between different study phases with a power of at least 80% (α level 5%). Individuals with poor metabolism or transport function phenotype were excluded from respective statistical comparisons. Additionally, to keep comparisons balanced, subjects lacking pharmacokinetic variable data in any study phase were excluded from the respective analyses. Prior to statistical analyses with IBM SPSS Statistics Version 29.0 for Windows (IBM Corporation, Armonk, NY), all pharmacokinetic variable values were logarithmically transformed. Pharmacokinetic variables except *T*_max_ were compared between different study phases by repeated‐measures analysis of variance with study phase as a within‐subjects factor. *T*_max_ values were compared with Wilcoxon signed‐rank test. Differences with *P* < 0.05 after the Bonferroni correction were considered statistically significant. The data are presented as geometrical mean values or geometric mean ratios with geometric CV or 90% confidence intervals (CI) if not otherwise indicated.

### Ethics statement

The Coordinating Ethics Committee of the Helsinki and Uusimaa Hospital District (record number HUS/1601/2021) and the Finnish Medicines Agency (EudraCT number 2020–003282‐19) gave approval for this study in August 2021 (conducted September 2021 to January 2022).

## RESULTS

### Participants

All 16 healthy volunteers, eight females and eight males, completed all study phases. The mean age (± standard deviation) of study participants was 23 ± 3 years, mean height 1.75 ± 0.09 m, mean weight 68.6 ± 9.8 kg, and mean body mass index 22.3 ± 2.2 kg/m^2^. Genotypes of CYP enzymes and transporters are described in **Table** **S3**.

### Addition of repaglinide into the Geneva cocktail

**Figure 1:**
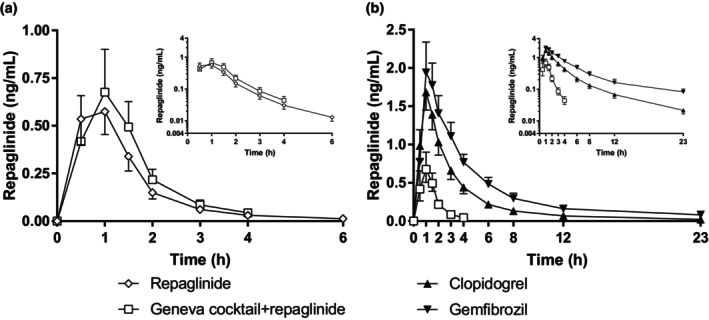
Repaglinide concentrations as geometric mean values ± 90% confidence intervals with and without the Geneva cocktail (**a**) and after clopidogrel pretreatment and gemfibrozil pretreatment compared with Geneva cocktail + repaglinide (**b**). Results are presented in two subfigures for clarity.

The Geneva cocktail increased the AUC_0‐23h_ of repaglinide 1.22‐fold (90% CI: 1.04–1.44, *P* = 0.033) compared with repaglinide alone but did not affect the *C*_max_, *T*_max_ or *t*_1/2_ of repaglinide (**Figure** **1a**, **Table** **2**, **Table** **S2**). The AUC_0‐12h_ and AUC_0‐23h_ values of the Geneva cocktail index drugs and their metabolites remained unaffected by the addition of repaglinide (**Table** **2**, **Table** **S2**).

**Table 2:** AUC_0‐12h_ and AUC_0‐23h_ values of index drugs and respective AUC ratios (AUC of CYP‐selective metabolite/AUC of index drug) in five study phases (I repaglinide, II Geneva cocktail, III Geneva cocktail + repaglinide (full cocktail), IV clopidogrel pretreatment followed by the full cocktail and V gemfibrozil pretreatment followed by the full cocktail) as geometric mean values (with geometric CV). Geometric mean ratios, GMR, (with 90% confidence intervals) compared with the respective control phase are indicated on the rows below each pharmacokinetic variable

	Repaglinide	Geneva cocktail	Geneva cocktail + repaglinide	Clopidogrel pretreatment	Gemfibrozil pretreatment
CYP1A2 (*N* = 16)					
Caffeine AUC_0‐12h_ (ng × h/mL)		6,250 (34.7%)	6,270 (36.0%)	6,960 (34.0%)	7,540 (32.0%)
GMR		Control	1.00 (0.86–1.17)		
GMR			Control	1.11 (0.90–1.37)	1.20* (1.03–1.40)
Paraxanthine/caffeine AUC_0‐12h_ ratio		0.54 (30.8%)	0.58 (23.4%)	0.57 (41.4%)	0.45 (34.3%)
GMR		Control	1.08 (0.95–1.22)		
GMR			Control	0.97 (0.82–1.16)	0.77** (0.69–0.86)
CYP2B6 (*N* = 15)^a^					
Bupropion AUC_0‐23h_ (ng × h/mL)		76.5 (33.4%)	72.8 (29.6%)	87.6 (30.0%)	61.4 (31.2%)
GMR		Control	0.95 (0.80–1.13)		
GMR			Control	1.20 (1.00–1.45)	0.84* (0.73–0.98)
OH‐bupropion/bupropion AUC_0‐23h_ ratio		7.46 (41.1%)	7.91 (37.5%)	2.07 (32.0%)	9.52 (39.5%)
GMR		Control	1.06 (0.83–1.35)		
GMR			Control	0.26** (0.20–0.35)	1.20 (0.91–1.59)
CYP2C8 (*N* = 16)					
Repaglinide AUC_0‐23h_ (ng × h/mL)	1.01 (53.7%)		1.23 (54.6%)	5.55 (42.0%)	9.21 (37.0%)
GMR	Control		1.22* (1.04–1.44)		
GMR			Control	4.51** (3.72–5.47)	7.49** (5.88–9.54)
CYP2C9 (*N* = 16)^b^					
Flurbiprofen AUC_0‐23h_ (ng × h/mL)		5,550 (26.9%)	5,270 (24.0%)	5,720 (21.6%)	5,770 (27.0%)
GMR		Control	0.95 (0.89–1.01)		
GMR			Control	1.09 (0.99–1.19)	1.10 (0.97–1.24)
4′‐OH‐flurbiprofen/flurbiprofen AUC_0‐23h_ ratio		0.040 (28.4%)	0.046 (40.5%)	0.042 (30.5%)	0.073 (29.8%)
GMR		Control	1.13 (0.95–1.34)		
GMR			Control	0.91 (0.80–1.03)	1.59** (1.32–1.92)
CYP2C19 (*N* = 15)^c^					
Omeprazole AUC_0‐23h_ (ng × h/mL)		177 (53.6%)	159 (64.1%)	214 (46.7%)	150 (62.8%)
GMR		Control	0.90 (0.70–1.14)		
GMR			Control	1.34* (1.09–1.66)	0.94 (0.70–1.27)
5′‐OH‐omeprazole/omeprazole AUC_0‐23h_ ratio		1.09 (39.4%)	1.15 (44.8%)	1.00 (42.5%)	1.69 (39.8%)
GMR		Control	1.05 (0.95–1.17)		
GMR			Control	0.87** (0.80–0.94)	1.47** (1.34–1.61)
CYP2D6 (*N* = 16)^d^					
Dextromethorphan AUC_0‐23h_ (ng × h/mL)		4.90 (117%)	5.54 (95.6%)	6.01 (118%)	6.07 (125%)
GMR		Control	1.13 (0.81–1.58)		
GMR			Control	1.09 (0.72–1.64)	1.10 (0.72–1.68)
Dextrorphan/dextromethorphan AUC_0‐23h_ ratio		1.65 (94.0%)	1.64 (105%)	1.61 (113%)	2.93 (109%)
GMR		Control	1.00 (0.80–1.24)		
GMR			Control	0.98 (0.70–1.38)	1.79** (1.23–2.59)
CYP3A4 (*N* = 16)^e^					
Midazolam AUC_0‐23h_ (ng × h/mL)		9.89 (42.1%)	8.43 (40.0%)	11.3 (38.0%)	8.06 (41.7%)
GMR		Control	0.85 (0.72–1.01)		
GMR			Control	1.34** (1.17–1.53)	0.96 (0.82–1.11)
1′‐OH‐midazolam/midazolam AUC_0‐23h_ ratio		0.36 (37.0%)	0.43 (38.2%)	0.45 (47.0%)	0.91 (42.6%)
GMR		Control	1.20* (1.04–1.39)		
GMR			Control	1.03 (0.88–1.20)	2.11** (1.87–2.38)

^a^ CYP2B6 phenotypes were the following: 1 rapid, 7 normal, 7 intermediate and 1 poor metabolizer, who was excluded from the statistical analyses regarding bupropion and hydroxybupropion.

^b^ CYP2C9 phenotypes were the following: 10 normal and 6 intermediate metabolizers.

^c^ CYP2C19 phenotypes were the following: 2 ultrarapid, 3 rapid, 5 normal and 6 intermediate metabolizers. One study participant was excluded due to delayed and erratic absorption of omeprazole in two study phases.

^d^ CYP2D6 phenotypes were the following: 1 ultrarapid, 10 normal, and 5 intermediate metabolizers.

^e^ CYP3A4 phenotypes were the following: 14 normal and 2 intermediate metabolizers.

*P < 0.05, **P < 0.01.

### Effect of clopidogrel

**Figure 2:**
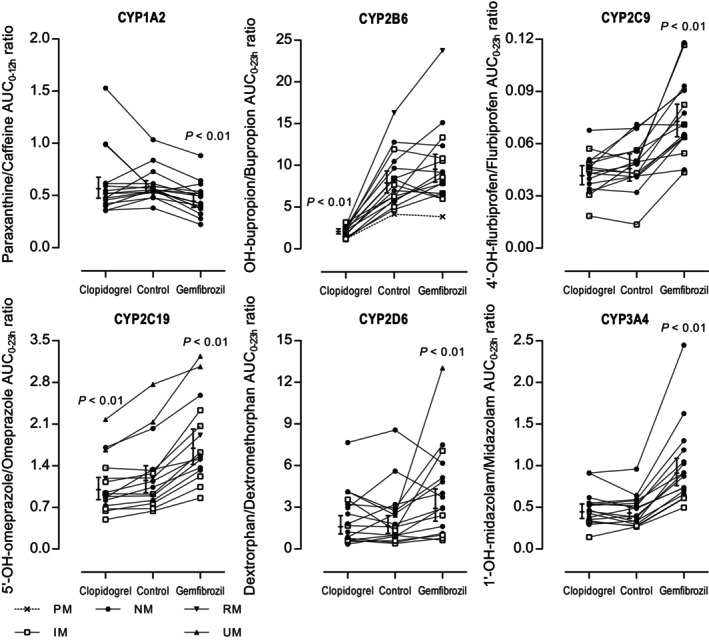
Individual AUC_0‐12h_ and AUC_0‐23h_ ratios of the Geneva cocktail index drugs in study phases with Geneva cocktail + repaglinide (full cocktail, control), clopidogrel pretreatment followed by the full cocktail and gemfibrozil pretreatment followed by the full cocktail represented with symbols for poor metabolizer (PM), intermediate metabolizer (IM), normal metabolizer (NM), rapid metabolizer (RM) and ultrarapid metabolizer (UM) CYP phenotypes. Geometric mean values ± 90% confidence intervals are expressed as horizontal lines with error bars. *P* < 0.05 are considered statistically significant.

**Figure 3:**
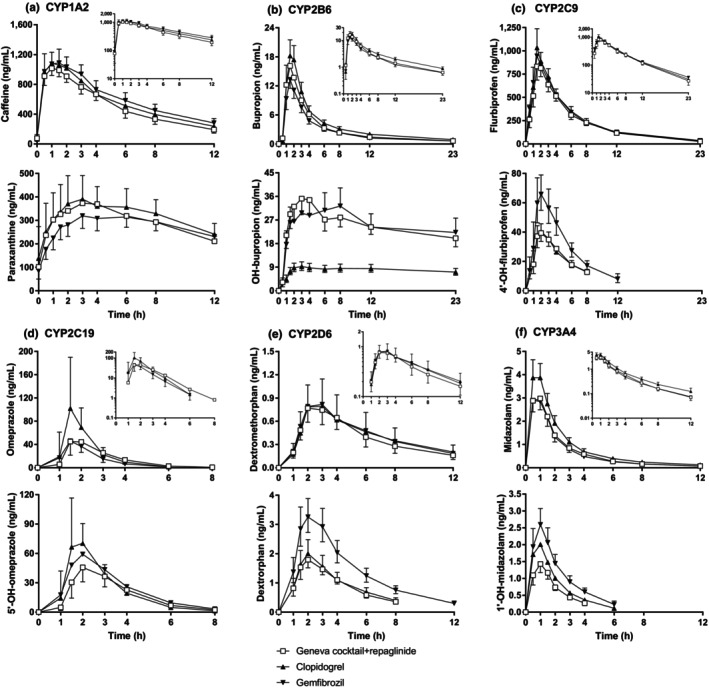
Concentrations of the Geneva cocktail index drugs and their metabolites for six CYP enzymes ((a) CYP1A2, (b) CYP2B6, (c) CYP2C9, (d) CYP2C19, (e) CYP2D6 and (f) CYP3A4) as geometric mean values ± 90% confidence intervals in study phases with Geneva cocktail + repaglinide (full cocktail), clopidogrel pretreatment followed by the full cocktail and gemfibrozil pretreatment followed by the full cocktail.

Clopidogrel increased the AUC_0‐23h_ and *t*_1/2_ of the CYP2C8 index substrate repaglinide 4.5‐fold (90% CI: 3.7–5.5, *P* < 0.01) and 2.5‐fold (90% CI: 2.0–3.0, *P* < 0.01), respectively (**Figure** **1** **b**, **Table** **2**, **Table** **S2**). After clopidogrel pretreatment, the hydroxybupropion/bupropion AUC_0‐23h_ ratio (CYP2B6 index) was decreased by 74% compared with control (90% CI: 65–80%, *P* < 0.01) (**Figures** **2** **and** **3**, **Table** **2**). While bupropion AUC_0‐23h_ did not increase significantly, that of hydroxybupropion was decreased markedly by clopidogrel (**Figure** **3**, **Table** **S2**). The 5′‐hydroxyomeprazole/omeprazole AUC_0‐23h_ ratio (CYP2C19 index) was decreased by 13% (90% CI: 6–20%, *P* < 0.01) and omeprazole concentrations and AUCs slightly increased by clopidogrel (**Figure** **2**, **Table** **S4**). Also, the *C*_max_ and AUC_0‐23h_ of the CYP3A4‐mediated metabolite of omeprazole, omeprazole sulfone, were increased by clopidogrel (*P* = 0.013 and *P* = 0.033, respectively) with no change in the omeprazole sulfone/omeprazole ratios (**Tables** **S2** **and** **S4**).

### Effect of gemfibrozil

Gemfibrozil increased the AUC_0‐23h_ and *t*_1/2_ of repaglinide 7.5‐fold (90% CI: 5.9–9.5, *P* < 0.01) and 3.0‐fold (90% CI: 2.5–3.6, *P* < 0.01), respectively (**Figure** **1** **b**, **Table** **2**, **Table** **S2**). The paraxanthine/caffeine AUC_0‐12h_ ratio (CYP1A2 index) was decreased by 23% (90% CI: 14–31%, *P* < 0.01) by gemfibrozil, while caffeine AUC_0‐12h_ was increased 1.20‐fold (1.03–1.40, *P* = 0.036) and *t*_1/2_ prolonged by 21% (8–35%, *P* < 0.01). The 4′‐hydroxyflurbiprofen/flurbiprofen (CYP2C9 index), 5′‐hydroxyomeprazole/omeprazole (CYP2C19 index), dextrorphan/dextromethorphan (CYP2D6 index) and 1′‐hydroxymidazolam/midazolam (CYP3A4 index) AUC_0‐23h_ ratios were increased by 1.59‐fold (90% CI: 1.32–1.92, *P* < 0.01), 1.47‐fold (90% CI: 1.34–1.61, *P* < 0.01), 1.79‐fold (90% CI: 1.23–2.59, *P* < 0.01), and 2.1‐fold (90% CI: 1.9–2.4, *P* < 0.01) by gemfibrozil, respectively (**Figure** **2**, **Table** **S4**). Similar effects were seen in the 2‐hour, 4‐hour, and AUC_0‐4h_ metabolite/index drug ratios (**Figure** **S1**, **Table** **S4**). Of note, the AUC_0‐23h_ values of 4′‐hydroxyflurbiprofen, 5′‐hydroxyomeprazole, dextrorphan, and 1′‐hydroxymidazolam were 1.75‐fold (90% CI: 1.45–2.11, *P* < 0.01), 1.38‐fold (90% CI: 1.07–1.79, *P* = 0.031), 1.96‐fold (90% CI: 1.69–2.26, *P* < 0.01), and 2.0‐fold (90% CI: 1.8–2.3, *P* < 0.01) higher during the gemfibrozil phase than during the control phase, respectively, while the AUC_0‐23h_ and *t*_1/2_ values of the respective index drugs remained unchanged (**Figure** **3**, **Table** **2**, **Table** **S2**).

### Pharmacokinetics of the pretreatment drugs

Clopidogrel and gemfibrozil concentrations are presented in **Table** **S5** and **Figure** **S2**.

### Biomarkers

When repaglinide only and Geneva cocktail only study phases were compared with the full cocktail phase, no difference in AUC_0‐3h_ of either GCDCA‐3G or GCDCA‐3S was found (**Table** **S6**). Clopidogrel did not change the C_0_ or AUC_0‐4h_ of the OATP1B1 biomarker GCDCA‐3G with statistical significance. On the other hand, gemfibrozil increased the C_0_ of GCDCA‐3G 1.56‐fold (90% CI 1.15–2.10, *P* = 0.015) and AUC_0‐4h_ 1.33‐fold (90% CI 1.07–1.65, *P* = 0.027) in individuals with either normal or decreased function phenotype of OATP1B1, whereas no effect was observed in individuals with the poor function phenotype of OATP1B1 (**Table** **3**). Neither clopidogrel nor gemfibrozil affected the C_0_ or AUC_0‐4h_ of OATP1B3 biomarker GCDCA‐3S (**Table** **3**). Gemfibrozil did not alter the CYP2D6‐dependent M430/solanidine and M444/solanidine metabolic ratios (**Table** **S7**).

**Table 3:** C_0_ concentrations and AUC_0‐4h_ values (based on concentrations from pretreatment drug administration at 8.00 am to sampling timepoint at 12 am on study days) of the OATP1B1 and OATP1B3 biomarkers GCDCA‐3G and GCDCA‐3S, respectively, during control, clopidogrel, and gemfibrozil phases expressed as geometric mean values (with geometric CV). Geometric mean ratios (GMR), and their 90% confidence intervals, compared with the respective control phase are indicated on the rows below each variable

	Geneva cocktail + repaglinide	Clopidogrel pretreatment	Gemfibrozil pretreatment
OATP1B1 normal/decreased function (*N* = 13)			
GCDCA‐3G C_0_ (ng/mL)	22.9 (60.0%)	21.7 (81.7%)	35.7 (82.5%)
GMR	Control	0.95 (0.71–1.25)	1.56 (1.15–2.10)
*P*‐value		> 0.99	0.015
GCDCA‐3G AUC_0‐4h_ (ng × h/mL)	88.6 (51.3%)	77.5 (69.9%)	118 (65.4%)
GMR	Control	0.87 (0.72–1.06)	1.33 (1.07–1.65)
*P*‐value		0.32	0.027
OATP1B1 poor function (*N* = 3)			
GCDCA‐3G C_0_ (ng/mL)	206 (73.8%)	238 (57.2%)	215 (79.1%)
GMR	Control	1.16 (0.72–1.86)	1.04 (0.64–1.71)
*P*‐value		0.63	> 0.99
GCDCA‐3G AUC_0‐4h_ (ng × h/mL)	755 (48.4%)	791 (56.1%)	809 (75.4%)
GMR	Control	1.05 (0.67–1.65)	1.07 (0.51–2.26)
*P*‐value		> 0.99	> 0.99
OATP1B3 (*N* = 16)			
GCDCA‐3S C_0_ (ng/mL)	50.8 (85.3%)	49.2 (69.3%)	65.5 (72.9%)
GMR	Control	0.97 (0.72–1.30)	1.29 (0.91–1.83)
*P*‐value		> 0.99	0.29
GCDCA‐3S AUC_0‐4h_ (ng × h/mL)	190 (67.4%)	146 (61.8%)	212 (46.9%)
GMR	Control	0.77 (0.59–1.00)	1.11 (0.83–1.49)
*P*‐value		0.095	0.91

Values for GCDCA‐3G are expressed for two different OATP1B1 phenotype groups. *P* < 0.05 is considered statistically significant.

### Safety

No adverse effects were reported during the study. Blood glucose levels were maintained > 3.5 mmol/L in all participants throughout the study. Mean blood glucose values were lowest 4 hours after the index drug administration but remained above 4.0 mmol/L (**Figure** **S3**). There was no need to serve additional carbohydrates to study participants.

## DISCUSSION

The results of our clinical study showed that a small 0.05 mg dose of the CYP2C8 index drug repaglinide had no effect on the pharmacokinetics of the Geneva cocktail CYP index drugs and was safe even when given with CYP2C8 inhibitors. Moreover, despite a slight increase in repaglinide AUC_0‐23h_ by the Geneva cocktail, the CYP2C8 inhibitors clopidogrel and gemfibrozil increased the AUC_0‐23h_ of repaglinide 4.5‐fold and 7.5‐fold, when the full cocktail was used, that is, to an equal degree as in previous studies with the same inhibitor doses.^13^, ^14^ Also, the known inhibitory effect of clopidogrel on CYP2B6 was captured accurately. Additionally, the plasma levels of sensitive OATP1B1 and 1B3 biomarkers were not altered between the repaglinide only, Geneva cocktail only, and full cocktail alone phases. Moreover, the CYP and OATP1B1 phenotype findings were in a good agreement with the respective genotypes (**Figure** **2**). Thus, the results indicate that the Geneva cocktail extended with a 0.05 mg dose of repaglinide can be used for simultaneous profiling of the activities of seven key CYP enzymes and two OATP1B biomarker activities. Slight inhibition of CYP1A2 and OATP1B1 and increase in the metabolic ratios of CYP2C9, CYP2C19, CYP2D6, and CYP3A4 were documented after gemfibrozil pretreatment.

Our study comprehensively profiled the effects of clopidogrel on CYP enzymes and OATP1B transporters. As expected,^24^, ^25^, ^26^ 300 mg clopidogrel caused a relatively strong effect on CYP2B6 indices, as hydroxybupropion/bupropion ratios decreased over 70%, which is in line with previous *in vitro* findings of mechanism‐based CYP2B6 inhibition by clopidogrel (**Figure** **3**, **Table** **S4**). The United States Food and Drug Administration (US FDA) has classified 75 mg dose of clopidogrel as a weak CYP2B6 inhibitor. However, in our study, 300 mg of clopidogrel caused a 74% decline in hydroxybupropion/bupropion AUC_0‐23h_ ratio indicating that a 300 mg clopidogrel dose could be used as a moderate CYP2B6 index inhibitor. In addition, clopidogrel slightly decreased the 5′‐hydroxyomeprazole/omeprazole AUC_0‐23h_ ratio, consistent with weak inhibition of CYP2C19 by clopidogrel, in good agreement with two previous clinical studies and *in vitro* data describing CYP2C19 inhibition by clopidogrel.^24^, ^27^, ^28^ Consequently, omeprazole metabolism shifted toward CYP3A4, whereby the *C*_max_ and AUC_0‐23h_ of omeprazole sulfone were increased after clopidogrel pretreatment (**Table** **S2**). Importantly, previous findings of lack of effect of clopidogrel on the pharmacokinetics of the sensitive OATP1B1 and CYP3A4 substrate simvastatin were corroborated as clopidogrel did not affect the metabolism of the sensitive CYP3A4 index drug midazolam or OATP1B biomarkers.^29^

An important secondary aim of this study was to profile the effects of gemfibrozil on CYP enzymes other than CYP2C8. Interestingly, gemfibrozil slightly reduced the paraxanthine/caffeine AUC_0‐12h_ ratio and increased the *t*_1/2_ and AUC_0‐12h_ of caffeine, consistent with weak CYP1A2 inhibition. Contrary to its 1‐O‐β‐glucuronide, gemfibrozil has been a weak CYP1A2 inhibitor *in vitro*.^26^, ^30^ Conversely, gemfibrozil increased the CYP2C9, CYP2C19, CYP2D6, and CYP3A4 index AUC_0‐23h_ ratios by about 50–100%, which could at first sight be interpreted as weak/moderate enzyme induction. However, further evaluation contradicts this impression as the concentrations or elimination half‐lives of these four index drugs were not decreased, while the index metabolite concentrations were elevated. Such findings are not consistent with accelerated metabolic clearance of the index drug but could be well explained by decreased elimination rates of the index metabolites.

Apart from strong CYP2C8 inhibitory effects of gemfibrozil and its glucuronide, their most potent *in vitro* CYP inhibitory effect has been against CYP2C9, while CYP2D6 inhibition has been very weak.^26^, ^30^ The present results are consistent with lack of any clinically meaningful CYP2C9 or CYP2D6 inhibition by gemfibrozil. Our results are equally in agreement with the limited and even contrasting effects of gemfibrozil on the pharmacokinetics of other CYP2C9 substrates, possibly explained by CYP2C8 inhibition and displacement from protein binding.^31^, ^32^, ^33^ Although dextrorphan/dextromethorphan metabolic ratios were increased after gemfibrozil pretreatment in our study, CYP2D6 is not known to be inducible. Consistent with the lack of induction, the M430/solanidine and M444/solanidine metabolic ratios, sensitive markers of CYP2D6 were not increased by gemfibrozil. Solanidine metabolic ratios are a promising alternative for dextromethorphan as a CYP2D6 index, but they require a diet containing solanidine. Accordingly, it may be useful to monitor solanidine metabolic ratios in addition to dextromethorphan to verify the mechanisms of possible interactions as demonstrated here.

We hypothesize that the mechanisms for the elevated concentrations of the index metabolites 4′‐hydroxyflurbiprofen, 5′‐hydroxyomeprazole, dextrorphan, and 1′‐hydroxymidazolam after gemfibrozil pretreatment involve inhibition of their further metabolism or transporter‐mediated excretion by gemfibrozil. UDP‐glucuronosyltransferases (UGTs) are likely involved in the further metabolism of these four index metabolites since they are converted to glucuronides.^34^, ^35^ In fact, UGT2B enzymes have been shown to metabolize 1′‐hydroxymidazolam and dextrorphan.^35^, ^36^ Interestingly, gemfibrozil can inhibit UGT‐mediated reactions *in vitro*.^37^, ^38^, ^39^ Moreover, an interplay between UGT enzymes and CYP2C8 has been suggested to affect drug–drug interactions caused by gemfibrozil.^40^ Accordingly, a potential explanation for the elevated index metabolite concentrations is impairment of their elimination by gemfibrozil either by direct inhibition of UGTs or by a complex hypothetical mechanism, where gemfibrozil inhibits a CYP2C8‐mediated elimination pathway of an unstable glucuronide metabolite, leading to accumulation of this glucuronide metabolite, which is then hydrolyzed back to the initial hydroxylated metabolite.^41^, ^42^ However, as clopidogrel, a moderate CYP2C8 inhibitor, had no effect on the concentrations of these four index metabolites, this kind of a complex solely CYP2C8‐mediated mechanism can be excluded. Another possibility is that gemfibrozil impaired transporter‐mediated elimination of the index metabolites or their glucuronides; apart from N‐glucuronides, particularly acyl glucuronides can be unstable, and therefore, impairment of their elimination could lead to elevation of the respective aglycone metabolites. For example, gemfibrozil seems to inhibit OAT3 that can mediate tubular excretion of some glucuronide metabolites.^43^ Nevertheless, further studies on the mechanisms of these novel gemfibrozil effects are required.

As gemfibrozil markedly increases the concentrations of OATP1B1 substrates like several statin acids and repaglinide,^16^, ^44^ we evaluated its effects on OATP1B1 and OATP1B3. We chose GCDCA‐3G and GCDCA‐3S as OATP1B1 and OATP1B3 biomarkers, respectively, because they have shown high selectivity and sensitivity toward these transporters, and apart from slight diurnal variation in their concentrations, they seem to have no weaknesses compared with alternative biomarkers.^18^, ^19^ Inhibition of both OATP1B1 and OATP1B3 by gemfibrozil have been demonstrated *in vitro*.^15^, ^45^ Clinically, different *SLCO1B1* genotypes have affected the degree of change in repaglinide pharmacokinetics caused by gemfibrozil.^46^ Accordingly, although alternative explanations exist for these interactions, gemfibrozil is thought to be a clinically significant OATP1B1 inhibitor.^44^ Based on our biomarker findings, gemfibrozil indeed slightly inhibits OATP1B1, but it does not affect OATP1B3 (**Table** **3**). *In vitro*, gemfibrozil 1‐O‐β‐glucuronide is a stronger OATP1B1 inhibitor than parent gemfibrozil.^16^ As the GCDCA‐3G C_0_ sample was taken 12 hours after the preceding gemfibrozil dose when residual gemfibrozil 1‐O‐β‐glucuronide concentrations were higher than gemfibrozil concentrations, our results may support the idea that OATP1B1 inhibition is mainly caused by gemfibrozil 1‐O‐β‐glucuronide (**Figure** **S2C,D**).

The advantages of our CYP activity phenotyping cocktail include oral administration of the index drugs, a clinically applicable venous blood sampling schedule, comprehensive inclusion of seven most important human drug‐metabolizing CYP enzymes, lack of significant drug–drug interactions between index drugs, and an excellent safety profile. In previous cocktails, common hindrances to clinical applicability have been caused by need to collect both blood and urine samples, mutual interactions between index drugs and adverse effects to study participants.^3^, ^8^, ^9^ In our study, a very low 0.05 mg dose of repaglinide was used and an hour later, breakfast was served to avoid hypoglycemia. With this procedure, no hypoglycemia was observed although repaglinide concentrations increased almost eightfold after gemfibrozil pretreatment. Repaglinide had no influence on the other index drugs and sensitively detected CYP2C8 inhibition. A disadvantage with the low dose was that repaglinide metabolite concentrations remained below the detection limit. Thus, CYP2C8 activity could only be assessed using repaglinide concentrations. The benefits of repaglinide are its sensitivity to CYP2C8 inhibition and its relatively short half‐life, allowing the detection of temporary changes in CYP2C8 activity.^47^, ^48^ On the other hand, an important caveat with repaglinide is that it is also an OATP1B1 substrate.^49^ Therefore, simultaneous use of a selective and sensitive biomarker of OATP1B1 activity, such as GCDCA‐3G, can be highly useful.^18^ Similarities between the recently advocated novel CYP2C8 index drug daprodustat and repaglinide include short half‐life and likely transport by OATP1B1.^50^ However, repaglinide is currently more accessible (daprodustat has no marketing authorization in Europe) and affordable and the clinical significance of detected CYP2C8 inhibition is more easily estimated due to abundance of previously published clinical trials with varying degrees of CYP2C8 inhibition.^13^, ^14^, ^47^, ^48^

The primary endpoint variables in CYP cocktail studies are typically either metabolic ratios at certain timepoints or AUC ratios.^2^, ^3^, ^4^, ^5^, ^6^, ^7^, ^9^ To reduce burden and increase the feasibility of a cocktail approach, limited sampling approaches are often used particularly in patient studies. For example, the 2‐hour metabolic ratio was the main phenotypic metric when the Geneva cocktail was used in a hospital study.^2^ Importantly, the changes in CYP index metabolite concentrations after gemfibrozil pretreatment in our study demonstrate that interpretation based on a single timepoint metabolic ratio can lead to uncertain or even false conclusions. This emphasizes the superiority of a more detailed AUC‐based pharmacokinetic evaluation to get mechanistic insights and plan further studies accordingly. Based on our data (**Tables** **S2** **and** **S4**), AUC_0‐4h_‐based evaluation could be a sufficient compromise if a truncated sample collection protocol is needed.

In conclusion, repaglinide did not have relevant interactions with the Geneva cocktail index drugs. The generated full seven‐in‐one CYP cocktail with biomarkers for two OATP1B activities, as well as any truncated form of the cocktail, are feasible methods in drug–drug interaction trials and other clinical trials or even clinical use, when CYP activity profiling is needed. In our trial, the index drug and biomarker data showed that clopidogrel caused moderate CYP2B6 and CYP2C8 inhibition and weak CYP2C19 inhibition, while gemfibrozil caused strong CYP2C8 inhibition and weak CYP1A2 and OATP1B1 inhibition, as well as unexpected increases in several CYP index metabolite concentrations. Overall, gemfibrozil is superior to clopidogrel as a CYP2C8 index inhibitor in terms of inhibition strength and selectivity. The discovered nonselective effects of gemfibrozil are noteworthy and indicate that caution is warranted when evaluating test inhibitor effects on the common CYP index substrates used in the present cocktail.

## FUNDING

This study was supported by grants from the Academy of Finland (Grant decision 325667, 2019), the Sigrid Jusélius Foundation (Grant number 8037; Helsinki, Finland), and by State Funding for University‐Level Health Research (TYH2019300 and TYH2021304); Hospital District of Helsinki and Uusimaa, Finland. Aurinsalo L received a personal grant from the Finnish Medical Foundation (Grant number: 4697, 2021; Helsinki, Finland).

## CONFLICT OF INTEREST

The authors declared no competing interests for this work.

## Author contributions

L.A., M.Ni., A.T., and J.T.B. designed the research; L.A., O.L.‐R., M.K., M.Ne., J.I.K., and J.T.B. performed the research; L.A., A.T., and J.T.B. analyzed the data; L.A., M.K., M.Ne., M.Ni., A.T., and J.T.B. wrote the manuscript.

## Supporting information


Data S1

## References

[1] Tornio, A. , Filppula, A.M. , Niemi, M. & Backman, J.T. Clinical studies on drug‐drug interactions involving metabolism and transport: methodology, pitfalls, and interpretation. Clin. Pharmacol. Ther. 105, 1345–1361 (2019). 30916389 10.1002/cpt.1435 PMC6563007

[2] Lenoir, C. *et al*. Impact of acute inflammation on cytochromes P450 activity assessed by the Geneva cocktail. Clin. Pharmacol. Ther. 109, 1668–1676 (2021). 33341941 10.1002/cpt.2146 PMC8247903

[3] Ryu, J.Y. *et al*. Development of the “Inje cocktail” for high‐throughput evaluation of five human cytochrome P450 isoforms in vivo. Clin. Pharmacol. Ther. 82, 531–540 (2007). 17392720 10.1038/sj.clpt.6100187

[4] Turpault, S. *et al*. Pharmacokinetic assessment of a five‐probe cocktail for CYPs 1A2, 2C9, 2C19, 2D6 and 3A. Br. J. Clin. Pharmacol. 68, 928–935 (2009). 20002088 10.1111/j.1365-2125.2009.03548.x PMC2810805

[5] Bosilkovska, M. *et al*. Evaluation of mutual drug‐drug interaction within Geneva cocktail for cytochrome P450 phenotyping using innovative dried blood sampling method. Basic Clin. Pharmacol. Toxicol. 119, 284–290 (2016). 27009433 10.1111/bcpt.12586

[6] Bosilkovska, M. *et al*. Geneva cocktail for cytochrome p450 and P‐glycoprotein activity assessment using dried blood spots. Clin. Pharmacol. Ther. 96, 349–359 (2014). 24722393 10.1038/clpt.2014.83 PMC4151019

[7] Donzelli, M. *et al*. The basel cocktail for simultaneous phenotyping of human cytochrome P450 isoforms in plasma, saliva and dried blood spots. Clin. Pharmacokinet. 53, 271–282 (2014). 24218006 10.1007/s40262-013-0115-0

[8] Bosilkovska, M. , Magliocco, G. , Desmeules, J. , Samer, C. & Daali, Y. Interaction between fexofenadine and CYP phenotyping probe drugs in Geneva cocktail. J. Pers. Med. 9, 45 (2019). 31581637 10.3390/jpm9040045 PMC6963818

[9] Fuhr, U. , Jetter, A. & Kirchheiner, J. Appropriate phenotyping procedures for drug metabolizing enzymes and transporters in humans and their simultaneous use in the “cocktail” approach. Clin. Pharmacol. Ther. 81, 270–283 (2007). 17259951 10.1038/sj.clpt.6100050

[10] Rollason, V. *et al*. Safety of the Geneva cocktail, a cytochrome P450 and P‐glycoprotein phenotyping cocktail, in healthy volunteers from three different geographic origins. Drug Saf. 43, 1181–1189 (2020). 32851583 10.1007/s40264-020-00983-8 PMC7575470

[11] Paludetto, M.N. , Kurkela, M. , Kahma, H. , Backman, J.T. , Niemi, M. & Filppula, A.M. Hydroxychloroquine is metabolized by cytochrome P450 2D6, 3A4, and 2C8, and inhibits cytochrome P450 2D6, while its metabolites also inhibit cytochrome P450 3A in vitro. Drug Metab. Dispos. 51, 293–305 (2023). 36446607 10.1124/dmd.122.001018

[12] Backman, J.T. , Filppula, A.M. , Niemi, M. & Neuvonen, P.J. Role of cytochrome P450 2C8 in drug metabolism and interactions. Pharmacol. Rev. 68, 168–241 (2016). 26721703 10.1124/pr.115.011411

[13] Tornio, A. *et al*. Glucuronidation converts clopidogrel to a strong time‐dependent inhibitor of CYP2C8: a phase II metabolite as a perpetrator of drug‐drug interactions. Clin. Pharmacol. Ther. 96, 498–507 (2014). 24971633 10.1038/clpt.2014.141

[14] Niemi, M. , Backman, J.T. , Neuvonen, M. & Neuvonen, P.J. Effects of gemfibrozil, itraconazole, and their combination on the pharmacokinetics and pharmacodynamics of repaglinide: potentially hazardous interaction between gemfibrozil and repaglinide. Diabetologia 46, 347–351 (2003). 12687332 10.1007/s00125-003-1034-7

[15] Shitara, Y. , Hirano, M. , Sato, H. & Sugiyama, Y. Gemfibrozil and its glucuronide inhibit the organic anion transporting polypeptide 2 (OATP2/OATP1B1:SLC21A6)‐mediated hepatic uptake and CYP2C8‐mediated metabolism of cerivastatin: analysis of the mechanism of the clinically relevant drug‐drug interaction between cerivastatin and gemfibrozil. J. Pharmacol. Exp. Ther. 311, 228–236 (2004). 15194707 10.1124/jpet.104.068536

[16] Tornio, A. , Neuvonen, P.J. , Niemi, M. & Backman, J.T. Role of gemfibrozil as an inhibitor of CYP2C8 and membrane transporters. Expert Opin. Drug Metab. Toxicol. 13, 83–95 (2017). 27548563 10.1080/17425255.2016.1227791

[17] Tamraz, B. *et al*. OATP1B1‐related drug‐drug and drug‐gene interactions as potential risk factors for cerivastatin‐induced rhabdomyolysis. Pharmacogenet. Genomics 23, 355–364 (2013). 23652407 10.1097/FPC.0b013e3283620c3b PMC3894639

[18] Neuvonen, M. *et al*. Identification of glycochenodeoxycholate 3‐O‐glucuronide and Glycodeoxycholate 3‐O‐glucuronide as highly sensitive and specific OATP1B1 biomarkers. Clin. Pharmacol. Ther. 109, 646–657 (2021). 32961594 10.1002/cpt.2053 PMC7983942

[19] Orozco, C.C. *et al*. Characterization of bile acid sulfate conjugates as substrates of human organic anion transporting polypeptides. Mol. Pharm. 20, 3020–3032 (2023). 37134201 10.1021/acs.molpharmaceut.3c00040

[20] Rago, B. , Tierney, B. , Rodrigues, A.D. , Holliman, C.L.H. & Ramanathan, R. A multiplex HRMS assay for quantifying selected human plasma bile acids as candidate OATP biomarkers. Bioanalysis 10, 645–657 (2018). 29749252 10.4155/bio-2017-0274

[21] Kiiski, J.I. *et al*. Solanidine is a sensitive and specific dietary biomarker for CYP2D6 activity. Hum. Genomics 18, 11 (2024). 38303026 10.1186/s40246-024-00579-8 PMC10835938

[22] Piha, M.O.W. *et al*. Candesartan has no clinically meaningful effect on the plasma concentrations of cytochrome P450 2C8 substrate repaglinide in humans. Drug Metab. Dispos. 52, 1388–1395 (2024). 39486868 10.1124/dmd.124.001798

[23] Litonius, K. *et al*. Value of pharmacogenetic testing assessed with real‐world drug utilization and genotype data. Clin. Pharmacol. Ther. 117, 278–288 (2025). 39365028 10.1002/cpt.3458 PMC11652815

[24] Richter, T. *et al*. Potent mechanism‐based inhibition of human CYP2B6 by clopidogrel and ticlopidine. J. Pharmacol. Exp. Ther. 308, 189–197 (2004). 14563790 10.1124/jpet.103.056127

[25] Turpeinen, M. , Tolonen, A. , Uusitalo, J. , Jalonen, J. , Pelkonen, O. & Laine, K. Effect of clopidogrel and ticlopidine on cytochrome P450 2B6 activity as measured by bupropion hydroxylation. Clin. Pharmacol. Ther. 77, 553–559 (2005). 15961986 10.1016/j.clpt.2005.02.010

[26] Kahma, H. *et al*. An automated cocktail method for in vitro assessment of direct and time‐dependent inhibition of nine major cytochrome P450 enzymes – application to establishing CYP2C8 inhibitor selectivity. Eur. J. Pharm. Sci. 162, 105810 (2021). 33753217 10.1016/j.ejps.2021.105810

[27] Ahmad, L. *et al*. Effect of clopidogrel on the hydroxylation and sulfoxidation of omeprazole: a single dose study in healthy human volunteers. EXCLI J. 16, 321–327 (2017). 28507476 10.17179/excli2016-658 PMC5427475

[28] Chen, B.L. *et al*. Clopidogrel inhibits CYP2C19‐dependent hydroxylation of omeprazole related to CYP2C19 genetic polymorphisms. J. Clin. Pharmacol. 49, 574–581 (2009). 19398604 10.1177/0091270009333016

[29] Itkonen, M.K. , Tornio, A. , Neuvonen, M. , Neuvonen, P.J. , Niemi, M. & Backman, J.T. Clopidogrel has no clinically meaningful effect on the pharmacokinetics of the organic anion transporting polypeptide 1B1 and cytochrome P450 3A4 substrate simvastatin. Drug Metab. Dispos. 43, 1655–1660 (2015). 26329790 10.1124/dmd.115.065938

[30] Wen, X. , Wang, J.S. , Backman, J.T. , Kivisto, K.T. & Neuvonen, P.J. Gemfibrozil is a potent inhibitor of human cytochrome P450 2C9. Drug Metab. Dispos. 29, 1359–1361 (2001). 11602509

[31] Niemi, M. , Neuvonen, P.J. & Kivisto, K.T. Effect of gemfibrozil on the pharmacokinetics and pharmacodynamics of glimepiride. Clin. Pharmacol. Ther. 70, 439–445 (2001). 11719730 10.1067/mcp.2001.119723

[32] Lilja, J.J. , Backman, J.T. & Neuvonen, P.J. Effect of gemfibrozil on the pharmacokinetics and pharmacodynamics of racemic warfarin in healthy subjects. Br. J. Clin. Pharmacol. 59, 433–439 (2005). 15801938 10.1111/j.1365-2125.2004.02323.x PMC1884811

[33] Tornio, A. , Niemi, M. , Neuvonen, P.J. & Backman, J.T. Stereoselective interaction between the CYP2C8 inhibitor gemfibrozil and racemic ibuprofen. Eur. J. Clin. Pharmacol. 63, 463–469 (2007). 17333159 10.1007/s00228-007-0273-9

[34] Duthaler, U. *et al*. The activity of members of the UDP‐glucuronosyltransferase subfamilies UGT1A and UGT2B is impaired in patients with liver cirrhosis. Clin. Pharmacokinet. 62, 1141–1155 (2023). 37328712 10.1007/s40262-023-01261-3 PMC10386950

[35] Rudesheim, S. , Selzer, D. , Fuhr, U. , Schwab, M. & Lehr, T. Physiologically‐based pharmacokinetic modeling of dextromethorphan to investigate interindividual variability within CYP2D6 activity score groups. CPT Pharmacometrics Syst. Pharmacol. 11, 494–511 (2022). 35257505 10.1002/psp4.12776 PMC9007601

[36] Seo, K.A. , Bae, S.K. , Choi, Y.K. , Choi, C.S. , Liu, K.H. & Shin, J.G. Metabolism of 1′‐ and 4‐hydroxymidazolam by glucuronide conjugation is largely mediated by UDP‐glucuronosyltransferases 1A4, 2B4, and 2B7. Drug Metab. Dispos. 38, 2007–2013 (2010). 20713656 10.1124/dmd.110.035295

[37] Prueksaritanont, T. , Tang, C. , Qiu, Y. , Mu, L. , Subramanian, R. & Lin, J.H. Effects of fibrates on metabolism of statins in human hepatocytes. Drug Metab. Dispos. 30, 1280–1287 (2002). 12386136 10.1124/dmd.30.11.1280

[38] Kato, Y. , Nakajima, M. , Oda, S. , Fukami, T. & Yokoi, T. Human UDP‐glucuronosyltransferase isoforms involved in haloperidol glucuronidation and quantitative estimation of their contribution. Drug Metab. Dispos. 40, 240–248 (2012). 22028316 10.1124/dmd.111.042150

[39] Gan, J. *et al*. Repaglinide‐gemfibrozil drug interaction: inhibition of repaglinide glucuronidation as a potential additional contributing mechanism. Br. J. Clin. Pharmacol. 70, 870–880 (2010). 21175442 10.1111/j.1365-2125.2010.03772.x PMC3014070

[40] Iga, K. & Kiriyama, A. Interplay of UDP‐glucuronosyltransferase and CYP2C8 for CYP2C8 mediated drug oxidation and its impact on drug‐drug interaction produced by standardized CYP2C8 inhibitors, clopidogrel and gemfibrozil. Clin. Pharmacokinet. 63, 43–56 (2024). 37921907 10.1007/s40262-023-01322-7

[41] Itkonen, M.K. , Tornio, A. , Neuvonen, M. , Neuvonen, P.J. , Niemi, M. & Backman, J.T. Clopidogrel and gemfibrozil strongly inhibit the CYP2C8‐dependent formation of 3‐Hydroxydesloratadine and increase desloratadine exposure in humans. Drug Metab. Dispos. 47, 377–385 (2019). 30630815 10.1124/dmd.118.084665

[42] Parkinson, A. , Kazmi, F. , Buckley, D.B. , Yerino, P. , Ogilvie, B.W. & Paris, B.L. System‐dependent outcomes during the evaluation of drug candidates as inhibitors of cytochrome P450 (CYP) and uridine diphosphate glucuronosyltransferase (UGT) enzymes: human hepatocytes versus liver microsomes versus recombinant enzymes. Drug Metab. Pharmacokinet. 25, 16–27 (2010). 20208386 10.2133/dmpk.25.16

[43] Watanabe, T. *et al*. Prediction of the overall renal tubular secretion and hepatic clearance of anionic drugs and a renal drug‐drug interaction involving organic anion transporter 3 in humans by in vitro uptake experiments. Drug Metab. Dispos. 39, 1031–1038 (2011). 21383204 10.1124/dmd.110.036129

[44] Backman, J.T. , Kyrklund, C. , Kivisto, K.T. , Wang, J.S. & Neuvonen, P.J. Plasma concentrations of active simvastatin acid are increased by gemfibrozil. Clin. Pharmacol. Ther. 68, 122–129 (2000). 10976543 10.1067/mcp.2000.108507

[45] Ho, R.H. *et al*. Drug and bile acid transporters in rosuvastatin hepatic uptake: function, expression, and pharmacogenetics. Gastroenterology 130, 1793–1806 (2006). 16697742 10.1053/j.gastro.2006.02.034

[46] Kalliokoski, A. , Backman, J.T. , Kurkinen, K.J. , Neuvonen, P.J. & Niemi, M. Effects of gemfibrozil and atorvastatin on the pharmacokinetics of repaglinide in relation to SLCO1B1 polymorphism. Clin. Pharmacol. Ther. 84, 488–496 (2008). 19238654 10.1038/clpt.2008.74

[47] Tornio, A. *et al*. The effect of gemfibrozil on repaglinide pharmacokinetics persists for at least 12 h after the dose: evidence for mechanism‐based inhibition of CYP2C8 in vivo. Clin. Pharmacol. Ther. 84, 403–411 (2008). 18388877 10.1038/clpt.2008.34

[48] Honkalammi, J. , Niemi, M. , Neuvonen, P.J. & Backman, J.T. Dose‐dependent interaction between gemfibrozil and repaglinide in humans: strong inhibition of CYP2C8 with subtherapeutic gemfibrozil doses. Drug Metab. Dispos. 39, 1977–1986 (2011). 21778352 10.1124/dmd.111.040931

[49] Niemi, M. *et al*. Polymorphic organic anion transporting polypeptide 1B1 is a major determinant of repaglinide pharmacokinetics. Clin. Pharmacol. Ther. 77, 468–478 (2005). 15961978 10.1016/j.clpt.2005.01.018

[50] Bi, Y.A. , Jordan, S. , King‐Ahmad, A. , West, M.A. & Varma, M.V.S. Mechanistic determinants of Daprodustat drug‐drug interactions and pharmacokinetics in hepatic dysfunction and chronic kidney disease: significance of OATP1B‐CYP2C8 interplay. Clin. Pharmacol. Ther. 115, 1336–1345 (2024). 38404228 10.1002/cpt.3215

