# Supplementary material for: A Phenotyping Tool for Seven Cytochrome P450 Enzymes and Two Transporters: Application to Examine the Effects of Clopidogrel and Gemfibrozil
Source: Clin Pharmacol Ther. 2025 Feb 21;117(6):1732–42. doi: 10.1002/cpt.3610 (PMC12087695; doi:10.1002/cpt.3610)
Supplement: Supplementary file 1 — Data S1 [file CPT-117-1732-s001.pdf]

SUPPLEMENTARY INFORMATION FOR

A Phenotyping Tool for Seven Cytochrome P450 Enzymes and Two Transporters: Application to Examine the Effects of Clopidogrel and Gemfibrozil

Aurinsalo, Laura[a,b,c]; Lapatto-Reiniluoto, Outi[a,b,c,,d]; Kurkela, Mika[a,b]; Neuvonen, Mikko[a,b]; Kiiski, Johanna I[a,b]; Niemi, Mikko[a,b,c]; Tornio, Aleksi[e,f*]; Backman, Janne T[a,b,c*]

[a]Department of Clinical Pharmacology, University of Helsinki, Helsinki, Finland

[b]Individualized Drug Therapy Research Program, Faculty of Medicine, University of Helsinki, Helsinki, Finland.

[c]Department of Clinical Pharmacology, HUS Diagnostic Center, Helsinki University Hospital, Helsinki, Finland

[d]HUS Pharmacy, Helsinki University Hospital, Helsinki, Finland

[e]Integrative Physiology and Pharmacology, Institute of Biomedicine, University of Turku, Turku, Finland

[f]Unit of Clinical Pharmacology, Turku University Hospital, Turku, Finland

*equal contribution

TABLE OF CONTENTS……………………………………………………………...……2

SUPPLEMENTARY METHODS……………………………………………………..3

Genotyping……………………………………………………………………………….3

SUPPLEMENTARY FIGURES…………………………………………………………….4

Figure S1………………………………………………………………………………4

Figure S2………………………………………………………………………………5

Figure S3………………………………………………………………………………6

SUPPLEMENTARY TABLES……………………………………………………...7

Table S1……………………………………………………………………………..7

Table S2………………………………………………………………………..………8

Table S3………………………………………………………………………………17

Table S4……………………………………………………………………..……..19

Table S5……………………………………………………………………..……..24

Table S6………………………………………………………………………………25

Table S7………………………………………………………………………………26

SUPPLEMENTARY METHODS

Genotyping

Genomic DNA was extracted from buffy coats prepared from EDTA-anticoagulated blood samples using the Maxwell 16 LEV Blood DNA Kit on a Maxwell 16 Research automated nucleic acid extraction system (Promega, Madison, WI). The samples were genotyped with an accredited clinical pharmacogenetic panel test available at the Genome Unit of the HUS Diagnostic Center (Helsinki University Hospital, Helsinki, Finland). Genotyping was carried out with massive parallel sequencing on the Ion GeneStudio™ S5 Prime System (Thermo Fisher Scientific, Waltham, MA) as described previously (Litonius, K., et al.). The panel covers clinically relevant variants in the *ABCG2*, *CYP2B6*, *CYP2C9*, *CYP2C19*, *CYP2D6*, *CYP3A5*, *CYP4F2*, *DPYD*, *NUDT15*, *SLCO1B1*, *TPMT*, and *VKORC1* genes. The genotypes were translated to phenotypes using standard methods (Litonius, K., et al.).

References:

Litonius, K., et al., Value of Pharmacogenetic Testing Assessed with Real-World Drug Utilization and Genotype Data. *Clin. Pharmacol. Ther.* **117(1)**, 278-288 (2025).

SUPPLEMENTARY FIGURES

Figure S1. Individual 2-hour metabolic ratios (2h MR) of the Geneva cocktail index drugs in study phases with Geneva cocktail + repaglinide (full cocktail, control), clopidogrel pretreatment followed by the full cocktail and gemfibrozil pretreatment followed by the full cocktail represented with symbols for poor metabolizer (PM), intermediate metabolizer (IM), normal metabolizer (NM), rapid metabolizer (RM) and ultrarapid metabolizer (UM) phenotypes for each CYP. Geometric mean values +/- 90% confidence intervals are shown as horizontal lines with error bars. *P*<0.05 are considered statistically significant.

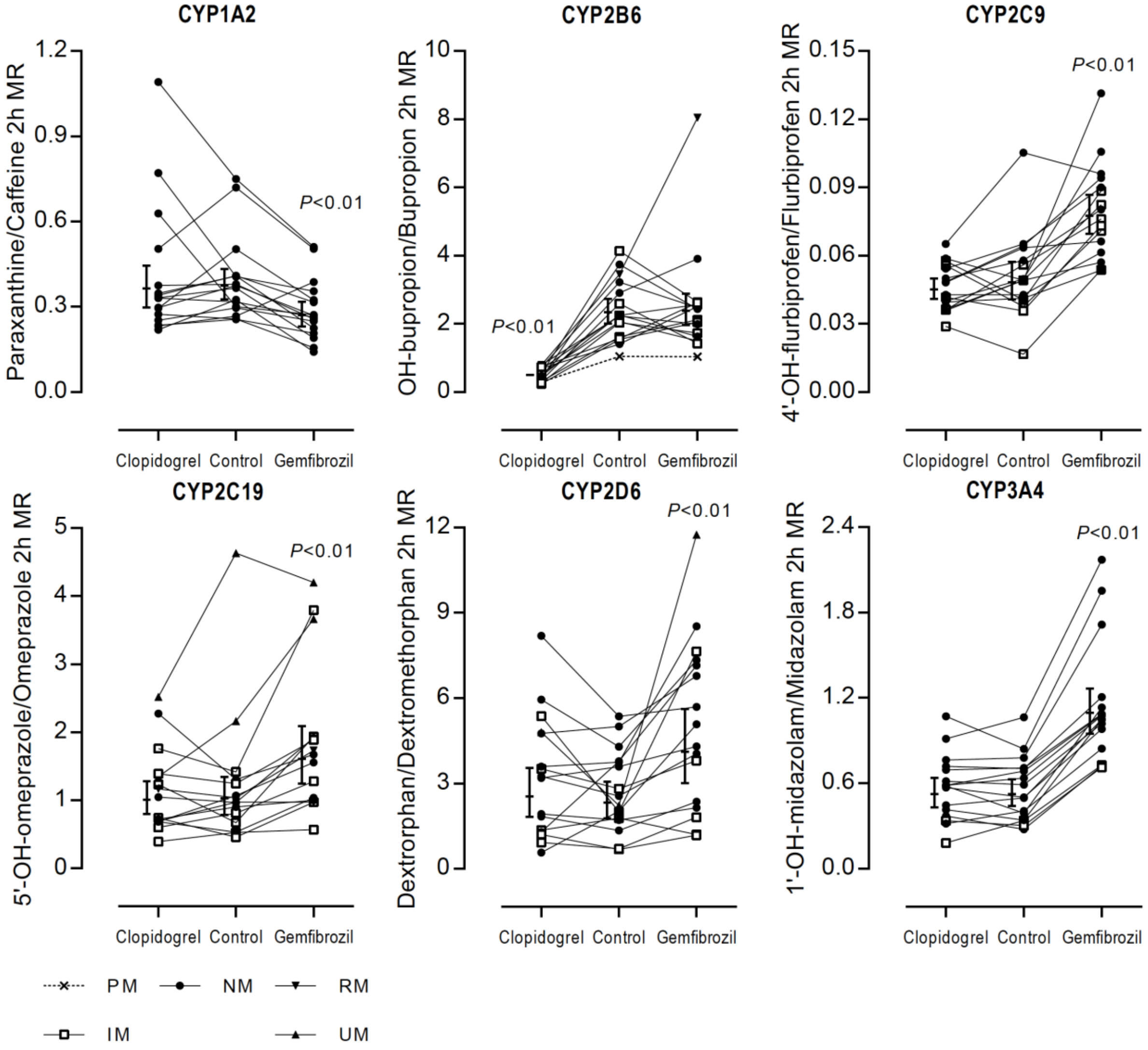

Figure S2. Concentrations of clopidogrel (A), its metabolites clopidogrel acyl-β-D-glucuronide and clopidogrel carboxylic acid (B), gemfibrozil (C) and its metabolite gemfibrozil 1-O-β-glucuronide (D) as geometric mean values +/- 90% confidence intervals. Time scale for subfigures A and B is from 8 a.m. when clopidogrel was administered until the next morning. For subfigures C and D, time scale is from 8 a.m. on study days when morning dose of gemfibrozil was administered until the last sampling time before the evening dose of gemfibrozil.

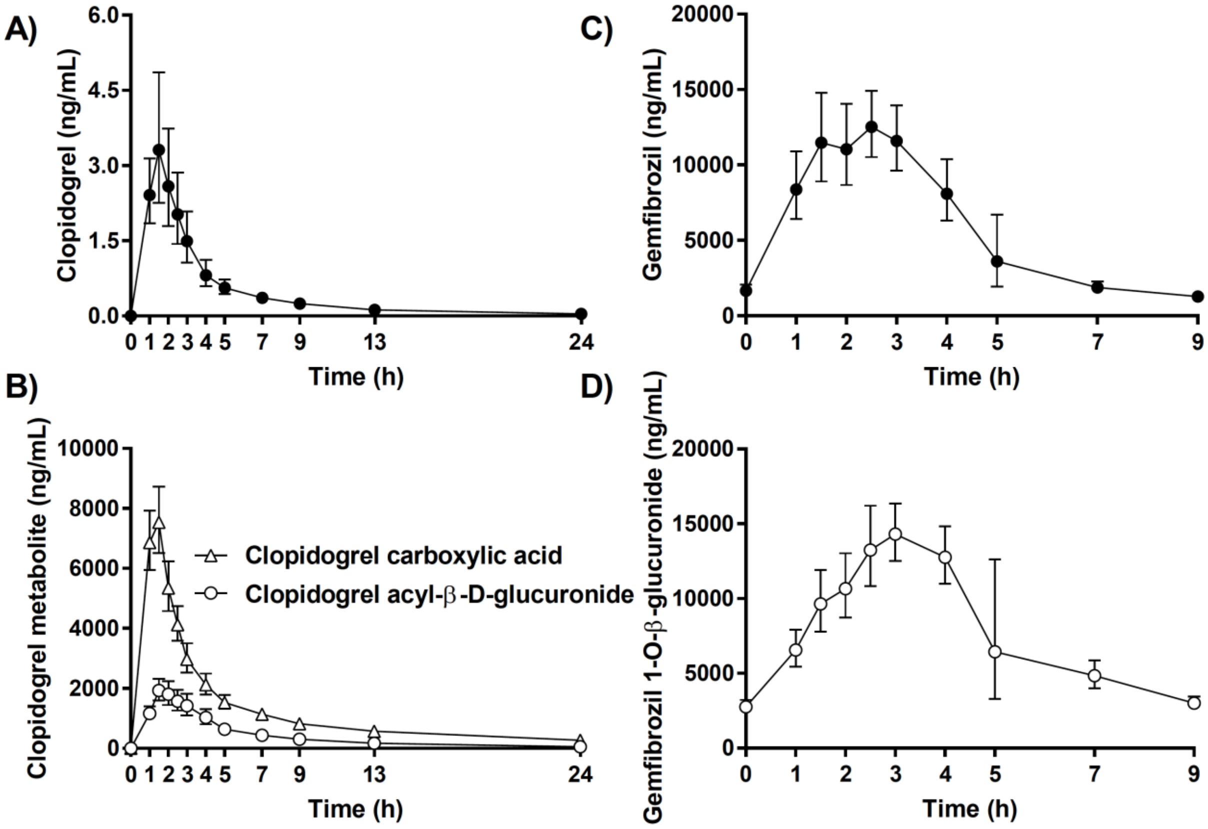

Figure S3. Mean blood glucose values (+/-S.D.) in different study phases with either repaglinide, Geneva cocktail, Geneva cocktail + repaglinide (full cocktail), clopidogrel pretreatment followed by the full cocktail or gemfibrozil pretreatment followed by the full cocktail. Standard meals served during study days are indicated with arrows.

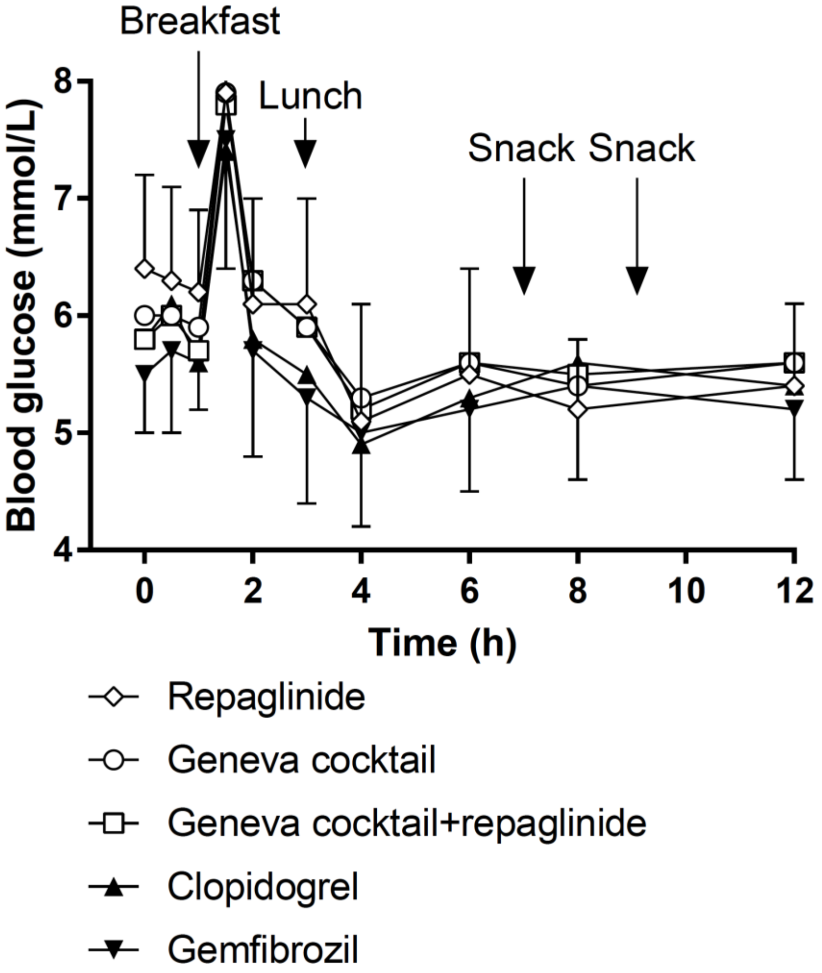

SUPPLEMENTARY TABLES

Table S1. Mass spectrometry parameters for index drugs, pretreatment drugs and their metabolites, and internal standards.

CYP	Index drugs, metabolites and Internal standards	MRM transition (*m/z*)	ESI +/-	CE	CXP
1A2	Caffeine	195.3 > 138.2	+	27	15
	Caffeine-d9	204.4 > 144.3	+	29	15
	Paraxanthine	181.2 > 124.2	+	27	13
2B6	Bupropion	240.2 > 184.2	+	17	17
	Bupropion-d9	249.3 > 185.3	+	19	21
	Hydroxybupropion	256.1 > 238.1	+	19	17
	Hydroxybupropion-d6	262.1 > 244.4	+	19	21
2C9	Flurbiprofen	198.9 > 198.9	-	-14	-19
	Flurbiprofen-d5	248.1 > 203.9	-	-14	-19
	4’-Hydroxyflurbiprofen	258.9 > 215	-	-14	-19
2C19	Omeprazole	346.3 > 198.1	+	15	23
	Omeprazole-d5	349.2 > 198.3	+	15	17
	5’-Hydroxyomeprazole	362.2 > 214.2	+	15	23
	5’-Hydroxyomeprazole-d3	365.1 > 214.3	+	17	21
2D6	Dextromethorphan	272.4 > 215.3	+	33	21
	Dextromethorphan-d3	275.3 > 215.4	+	33	11
	Dextrorphan	258.2 > 199.1	+	37	17
	Dextrorphan-d3	261.2 > 157.4	+	51	17
3A4	Midazolam	326.3 > 291.2	+	37	19
	Midazolam-d6	332.3 > 297.2	+	37	19
	1’-Hydroxymidazolam	342.1 > 324.3	+	34	23
	1’-Hydroxymidazolam-d4	346.1 > 203.3	+	33	17
	Omeprazole sulfone	362.2 > 150.2	+	33	21
	Omeprazole-d3 sulfone	365.2 > 150.3	+	35	17
Pretreatment drugs and metabolites		MRM transition (*m/z*)	ESI +/-	CE	CXP
	Clopidogrel	322.3 > 212.4	+	21	19
	Clopidogrel-d9	331.3 > 219.4	+	23	21
	Clopidogrel carboxylic acid	308.2 > 198	+	23	14
	Clopidogrel-d6 carboxylic acid	314 > 204	+	23	14
	Clopidogrel acyl-β-D-glucuronide	484.2 > 308.2	+	19	12
	Gemfibrozil	246 > 120.9	-	-20	-5
	Gemfibrozil-d6	255 > 120.9	-	-20	-5
	Gemfibrozil 1-O-glucuronide	425.2 > 121	-	-42	-21
	Gemfibrozil-d6 1-O-glucuronide	431.2 > 120.9	-	-42	-5

MRM, multiple reaction monitoring, ESI, electro spray ionization, CE, collision energy, CXP, collision cell exit potential

Table S2. Pharmacokinetic values of index drugs and their metabolites in five study phases (I repaglinide, II Geneva cocktail, III Geneva cocktail + repaglinide, IV clopidogrel pretreatment and V gemfibrozil pretreatment) as geometric mean values (with geometric CV) except for T_{max} which is given as median with range. Geometric mean ratios (GMR) (90% confidence interval) compared to the respective control phase are indicated on the rows below each pharmacokinetic variable.

	Repaglinide	Geneva cocktail	Geneva cocktail + repaglinide	Clopidogrel pretreatment	Gemfibrozil pretreatment
**Caffeine (N=16)**					
C_{max} (ng/mL)		1130 (24.3%)	1100 (22.4%)	1180 (25.8%)	1250 (26.9%)
GMR		*Control*	0.97 (0.87-1.09)		
GMR			*Control*	1.07 (0.90-1.29)	1.14 (0.98-1.32)
T_{max} (h)		1.0 (0.5-2.0)	1.3 (0.5-2.0)	1.0 (0.5-3.0)	1.3 (0.5-2.0)
AUC_{0-12h} (ng×h/mL)		6250 (34.7%)	6270 (36.0%)	6960 (34.0%)	7540 (32.0%)
GMR		*Control*	1.00 (0.86-1.17)		
GMR			*Control*	1.11 (0.90-1.37)	1.20* (1.03-1.40)
$AUC_{0-\infty}$ (ng×h/mL)		7830 (46.0%)	7800 (50.7%)	8970 (46.6%)	10 100 (42.3%)
GMR		*Control*	1.00 (0.86-1.16)		
GMR			*Control*	1.15 (0.90-1.46)	1.30** (1.11-1.52)
$t_{1/2}$ (h)		4.65 (38.9%)	4.62 (37.1%)	5.05 (35.9%)	5.57 (36.6%)
GMR		*Control*	0.99 (0.91-1.08)		
GMR			*Control*	1.09 (0.98-1.22)	1.21** (1.08-1.35)
**Paraxanthine (N=16)**					
C_{max} (ng/mL)		374 (45.8%)	405 (31.7%)	436 (66.6%)	358 (43.1%)
GMR		*Control*	1.09 (0.91-1.29)		
GMR			*Control*	1.08 (0.82-1.41)	0.88 (0.71-1.09)
T_{max} (h)		4.0 (0.5-12.0)	3.0 (1.0-8.0)	3.0 (0.0-8.0)	5.0* (1.0-12.0)
AUC_{0-12h} (ng×h/mL)		3370 (44.1%)	3640 (31.3%)	3940 (51.6%)	3360 (36.9%)
GMR		*Control*	1.08 (0.89-1.32)		
GMR			*Control*	1.08 (0.85-1.37)	0.92 (0.74-1.15)

**Paraxanthine (N=16)**					
$AUC_{0-\infty}$ (ng×h/mL)		7220 (80.0%)	7750 (82.5%)	8930 (57.5%)	9160 (49.1%)
GMR		*Control*	1.07 (0.84-1.37)		
GMR			*Control*	1.15 (0.87-1.53)	1.18 (0.90-1.56)
$t_{1/2}$ (h)		11.0 (77.9%)	11.5 (77.9%)	12.8 (62.7%)	15.9 (63.1%)
GMR		*Control*	1.04 (0.84-1.28)		
GMR			*Control*	1.11 (0.87-1.41)	1.38* (1.08-1.77)
**Bupropion[1] (N=15)**					
C_{max} (ng/mL)		20.0 (40.7%)	18.0 (38.3%)	19.9 (37.6%)	14.6 (29.8%)
GMR		*Control*	0.90 (0.72-1.12)		
GMR			*Control*	1.11 (0.94-1.30)	0.81* (0.69-0.95)
T_{max} (h)		1.5 (1.0-3.0)	1.5 (1.0-2.0)	1.5 (1.0-2.0)	1.5 (1.0-2.0)
AUC_{0-23h} (ng×h/mL)		76.5 (33.4%)	72.8 (29.6%)	87.6 (30.0%)	61.4 (31.2%)
GMR		*Control*	0.95 (0.80-1.13)		
GMR			*Control*	1.20 (1.00-1.45)	0.84* (0.73-0.98)
$AUC_{0-\infty}$ (ng×h/mL)		84.7 (34.2%)	80.5 (29.2%)	98.1 (29.6%)	68.8 (34.2%)
GMR		*Control*	0.95 (0.80-1.12)		
GMR			*Control*	1.22 (1.02-1.45)	0.85 (0.74-0.99)
$t_{1/2}$ (h)		7.89 (19.9%)	7.50 (24.0%)	7.92 (14.7%)	7.67 (17.0%)
GMR		*Control*	0.95 (0.82-1.10)		
GMR			*Control*	1.06 (0.91-1.22)	1.02 (0.92-1.14)
**OH-bupropion[1] (N=15)**					
C_{max} (ng/mL)		36.9 (38.6%)	37.6 (37.0%)	10.3 (39.9%)	34.1 (44.6%)
GMR		*Control*	1.02 (0.85-1.22)		
GMR			*Control*	0.27** (0.22-0.34)	0.91 (0.74-1.11)
T_{max} (h)		3.0 (2.0-4.0)	3.0 (1.5-8.0)	4.0 (1.5-12.0)	6.0 (1.0-12.0)

**OH-bupropion[1] (N=15)**					
$AUC_{0\text{-}23h}$ (ng×h/mL)		571 (35.3%)	575 (30.9%)	181 (37.6%)	585 (43.5%)
GMR		*Control*	1.01 (0.84-1.21)		
GMR			*Control*	0.31** (0.26-0.38)	1.02 (0.85-1.21)
$AUC_{0\text{-}\infty}$ (ng×h/mL) (N=11)[2]		1200 (44.1%)	1170 (42.3%)	499 (45.3%)	1380 (41.2%)
GMR		*Control*	0.98 (0.61-1.56)		
GMR			*Control*	0.43** (0.31-0.59)	1.18* (1.02-1.36)
$t_{1/2}$ (h) (N=11)[2]		26.1 (40.1%)	23.2 (46.0%)	34.6 (42.9%)	29.4 (51.7%)
GMR		*Control*	0.89 (0.59-1.33)		
GMR			*Control*	1.49* (1.15-1.93)	1.26 (0.95-1.67)
**Repaglinide (N=16)**					
C_{max} (ng/mL)	0.701 (51.1%)		0.806 (60.7%)	1.69 (33.6%)	2.08 (36.3%)
GMR	*Control*		1.15 (0.89-1.49)		
GMR			*Control*	2.10** (1.68-2.63)	2.58** (1.93-3.45)
T_{max} (h)	0.5 (0.5-1.0)		1.0 (0.5-1.5)	1.0 (0.5-1.5)	1.0* (1.0-1.5)
$AUC_{0\text{-}4h}$ (ng×h/mL)	0.936 (51.8%)		1.13 (54.3%)	3.70 (36.4%)	4.93 (33.7%)
GMR	*Control*		1.21* (1.03-1.42)		
GMR			*Control*	3.28** (2.71-3.97)	4.37** (3.43-5.56)
$AUC_{0\text{-}23h}$ (ng×h/mL)	1.01 (53.7%)		1.23 (54.6%)	5.55 (42.0%)	9.21 (37.0%)
GMR	*Control*		1.22* (1.04-1.44)		
GMR			*Control*	4.51** (3.72-5.47)	7.49** (5.88-9.54)
$AUC_{0\text{-}\infty}$ (ng×h/mL)	1.01 (53.6%)		1.23 (54.4%)	5.65 (42.4%)	9.64 (38.1%)
GMR	*Control*		1.22* (1.04-1.43)		
GMR			*Control*	4.59** (3.78-5.58)	7.84** (6.15-10.0)
$t_{1/2}$ (h)	1.07 (27.8%)		1.13 (22.5%)	2.78 (13.3%)	3.35 (18.9%)
GMR	*Control*		1.06 (0.90-1.24)		
GMR			*Control*	2.46** (2.05-2.95)	2.96** (2.46-3.55)

**Flurbiprofen[3] (N=16)**					
C_{max} (ng/mL)		1090 (41.4%)	976 (29.0%)	1180 (21.7%)	1110 (29.7%)
GMR		*Control*	0.90 (0.72-1.11)		
GMR			*Control*	1.21* (1.03-1.43)	1.14 (0.93-1.39)
T_{max} (h)		1.5 (1.0-2.0)	1.5 (1.0-6.0)	1.5 (0.5-3.0)	1.5 (0.5-4.0)
$AUC_{0\text{-}23h}$ (ng×h/mL)		5550 (26.9%)	5270 (24.0%)	5720 (21.6%)	5770 (27.0%)
GMR		*Control*	0.95 (0.89-1.01)		
GMR			*Control*	1.09 (0.99-1.19)	1.10 (0.97-1.24)
$AUC_{0\text{-}\infty}$ (ng×h/mL)		5910 (26.7%)	5480 (26.5%)	5940 (23.9%)	6080 (29.2%)
GMR		*Control*	0.93 (0.86-1.00)		
GMR			*Control*	1.08 (0.99-1.19)	1.11 (0.97-1.27)
$t_{1/2}$ (h)		4.82 (23.6%)	4.53 (26.1%)	4.35 (24.6%)	4.61 (24.9%)
GMR		*Control*	0.94 (0.84-1.06)		
GMR			*Control*	0.96 (0.88-1.05)	1.02 (0.93-1.12)
**4'-OH-flurbiprofen[3] (N=16)**					
C_{max} (ng/mL)		46.4 (38.4%)	45.7 (36.5%)	46.9 (21.4%)	78.0 (39.2%)
GMR		*Control*	0.99 (0.76-1.27)		
GMR			*Control*	1.03 (0.88-1.20)	1.71** (1.35-2.15)
T_{max} (h)		1.8 (1.0-3.0)	1.5 (1.0-4.0)	2.0 (1.0-3.0)	1.8 (1.0-4.0)
$AUC_{0\text{-}23h}$ (ng×h/mL)		224 (26.6%)	241 (30.3%)	238 (28.3%)	420 (38.7%)
GMR		*Control*	1.07 (0.90-1.27)		
GMR			*Control*	0.99 (0.87-1.13)	1.75** (1.45-2.11)
$AUC_{0\text{-}\infty}$ (ng×h/mL)		263 (23.2%)	266 (26.3%)	269 (22.2%)	432 (34.3%)
GMR		*Control*	1.01 (0.85-1.20)		
GMR			*Control*	1.01 (0.87-1.17)	1.62** (1.38-1.91)

**4'-OH-flurbiprofen[3] (N=16)**					
$t_{1/2}$ (h)		3.44 (34.8%)	3.34 (24.3%)	3.59 (28.3%)	3.01 (20.9%)
GMR		*Control*	0.97 (0.78-1.21)		
GMR			*Control*	1.07 (0.89-1.29)	0.90 (0.77-1.06)
**Omeprazole[4,5,6] (N=15)**					
C_{max} (ng/mL)		144 (88.5%)	89.3 (107%)	167 (38.4%)	98.0 (99.5%)
GMR		*Control*	0.62 (0.38-1.01)		
GMR			*Control*	1.87** (1.24-2.83)	1.10 (0.61-1.97)
T_{max} (h)		1.5 (1.0-2.0)	2.0 (1.0-6.0)	1.5 (1.0-3.0)	1.5 (0.5-4.0)
AUC_{0-23h} (ng×h/mL)		177 (53.6%)	159 (64.1%)	214 (46.7%)	150 (62.8%)
GMR		*Control*	0.90 (0.70-1.14)		
GMR			*Control*	1.34* (1.09-1.66)	0.94 (0.70-1.27)
$AUC_{0-\infty}$ (ng×h/mL)		177 (54.0%)	158 (64.8%)	214 (46.6%)	149 (62.9%)
GMR		*Control*	0.89 (0.70-1.14)		
GMR			*Control*	1.35* (1.09-1.68)	0.95 (0.70-1.28)
$t_{1/2}$ (h)		0.76 (53.9%)	0.85 (32.9%)	0.69 (30.9%)	0.78 (59.9%)
GMR		*Control*	1.11 (0.88-1.40)		
GMR			*Control*	0.81* (0.68-0.96)	0.92 (0.69-1.23)
**5'-OH-omeprazole[4,5] (N=15)**					
C_{max} (ng/mL)		99.8 (87.9%)	70.8 (74.1%)	115 (23.8%)	104 (76.9%)
GMR		*Control*	0.71 (0.46-1.09)		
GMR			*Control*	1.63* (1.16-2.30)	1.48 (0.86-2.53)
T_{max} (h)		1.5 (1.0-2.0)	2.0 (1.0-8.0)	1.5 (1.0-3.0)	1.5 (1.0-4.0)
AUC_{0-23h} (ng×h/mL)		194 (35.7%)	183 (34.1%)	213 (14.6%)	253 (37.6%)
GMR		*Control*	0.95 (0.79-1.14)		
GMR			*Control*	1.16 (0.97-1.39)	1.38* (1.07-1.79)

**5’-OH-omeprazole[4,5] (N=15)**					
$AUC_{0-\infty}$ (ng×h/mL)		193 (36.1%)	181 (35.7%)	212 (14.5%)	253 (36.9%)
GMR		*Control*	0.94 (0.77-1.14)		
GMR			*Control*	1.17 (0.97-1.41)	1.40* (1.08-1.81)
$t_{1/2}$ (h)		1.17 (36.9%)	1.18 (24.8%)	1.04 (15.5%)	1.34 (32.6%)
GMR		*Control*	1.01 (0.88-1.16)		
GMR			*Control*	0.88 (0.78-0.99)	1.14 (0.94-1.37)
**Omeprazole sulfone[5,6] (N=15)**					
C_{max} (ng/mL)		35.3 (93.5%)	29.7 (64.1%)	51.2 (38.3%)	41.8 (88.0%)
GMR		*Control*	0.84 (0.54-1.31)		
GMR			*Control*	1.72* (1.18-2.51)	1.41 (0.85-2.32)
T_{max} (h)		1.50 (1.00-3.00)	2.00 (1.00-8.00)	1.50 (1.00-3.00)	1.50 (1.00-4.00)
AUC_{0-23h} (ng×h/mL)		113 (78.3%)	112 (57.5%)	156 (58.9%)	124 (73.3%)
GMR		*Control*	0.99 (0.75-1.32)		
GMR			*Control*	1.40* (1.07-1.82)	1.11 (0.82-1.51)
$AUC_{0-\infty}$ (ng×h/mL)		111 (78.3%)	110 (58.5%)	154 (59.2%)	122 (73.9%)
GMR		*Control*	0.99 (0.74-1.31)		
GMR			*Control*	1.40* (1.06-1.85)	1.11 (0.81-1.52)
$t_{1/2}$ (h)		1.90 (40.4%)	1.82 (34.6%)	1.88 (34.8%)	1.64 (34.2%)
GMR		*Control*	0.96 (0.83-1.11)		
GMR			*Control*	1.03 (0.93-1.14)	0.90 (0.79-1.03)
**Dextromethorphan[7] (N=16)**					
C_{max} (ng/mL)		0.745 (85.6%)	0.896 (66.6%)	0.858 (85.5%)	0.898 (84.3%)
GMR		*Control*	1.20 (0.84-1.72)		
GMR			*Control*	0.96 (0.66-1.40)	1.00 (0.71-1.42)
T_{max} (h)		2.0 (2.0-4.0)	2.0 (1.0-4.0)	2.0 (2.0-3.0)	2.5 (1.5-4.0)

**Dextromethorphan[7] (N=16)**					
AUC_{0-23h} (ng×h/mL)		4.90 (117%)	5.54 (95.6%)	6.01 (118%)	6.07 (125%)
GMR		*Control*	1.13 (0.81-1.58)		
GMR			*Control*	1.09 (0.72-1.64)	1.10 (0.72-1.68)
$AUC_{0-\infty}$ (ng×h/mL)		5.58 (110%)	6.52 (77.4%)	6.68 (115%)	6.82 (119%)
GMR		*Control*	1.17 (0.84-1.63)		
GMR			*Control*	1.02 (0.70-1.50)	1.05 (0.72-1.52)
$t_{1/2}$ (h)		4.24 (35.1%)	4.38 (38.3%)	4.61 (33.4%)	4.38 (31.8%)
GMR		*Control*	1.03 (0.85-1.26)		
GMR			*Control*	1.05 (0.88-1.27)	1.00 (0.80-1.25)
**Dextrorphan[7] (N=16)**					
C_{max} (ng/mL)		1.82 (38.5%)	1.98 (38.7%)	2.05 (52.3%)	3.63 (41.3%)
GMR		*Control*	1.09 (0.84-1.42)		
GMR			*Control*	1.04 (0.85-1.26)	1.83** (1.60-2.09)
T_{max} (h)		2.0 (1.5-2.0)	1.5 (1.0-3.0)	2.0 (1.0-3.0)	2.0 (1.5-3.0)
AUC_{0-23h} (ng×h/mL)		8.07 (42.2%)	9.09 (35.1%)	9.66 (49.1%)	17.8 (42.1%)
GMR		*Control*	1.13 (0.92-1.38)		
GMR			*Control*	1.06 (0.90-1.25)	1.96** (1.69-2.26)
$AUC_{0-\infty}$ (ng×h/mL)		8.35 (35.4%)	9.35 (32.5%)	10.0 (45.1%)	17.8 (40.5%)
GMR		*Control*	1.12 (0.92-1.36)		
GMR			*Control*	1.07 (0.91-1.27)	1.91** (1.65-2.20)
$t_{1/2}$ (h)		2.50 (15.1%)	2.52 (23.6%)	2.68 (22.9%)	2.77 (17.6%)
GMR		*Control*	1.01 (0.91-1.12)		
GMR			*Control*	1.06 (0.98-1.16)	1.10 (0.96-1.26)
**Midazolam[6] (N=16)**					
C_{max} (ng/mL)		4.03 (36.4%)	3.32 (37.4%)	4.33 (33.7%)	3.25 (36.6%)
GMR		*Control*	0.82* (0.69-0.97)		
GMR			*Control*	1.31** (1.13-1.50)	0.98 (0.82-1.17)

**Midazolam[6] (N=16)**					
T_{max} (h)		0.5 (0.5-1.0)	1.0 (0.5-1.5)	1.0 (0.5-1.0)	0.5 (0.5-1.0)
AUC_{0-23h} (ng×h/mL)		9.89 (42.1%)	8.43 (40.0%)	11.3 (38.0%)	8.06 (41.7%)
GMR		*Control*	0.85 (0.72-1.01)		
GMR			*Control*	1.34** (1.17-1.53)	0.96 (0.82-1.11)
$AUC_{0-\infty}$ (ng×h/mL)		9.82 (43.1%)	8.40 (39.9%)	11.2 (39.3%)	7.98 (40.8%)
GMR		*Control*	0.85 (0.73-1.00)		
GMR			*Control*	1.34** (1.17-1.53)	0.95 (0.83-1.09)
$t_{1/2}$ (h)		2.29 (28.8%)	2.16 (28.5%)	2.40 (29.4%)	2.29 (23.5%)
GMR		*Control*	0.94 (0.84-1.05)		
GMR			*Control*	1.12 (1.01-1.23)	1.06 (0.99-1.14)
**1'-OH-midazolam[6] (N=16)**					
C_{max} (ng/mL)		1.48 (40.9%)	1.56 (39.7%)	2.14 (42.5%)	2.83 (38.7%)
GMR		*Control*	1.05 (0.91-1.23)		
GMR			*Control*	1.37** (1.16-1.62)	1.81** (1.57-2.09)
T_{max} (h)		1.0 (0.5-1.0)	1.0 (0.5-2.0)	1.0 (0.5-1.0)	1.0 (0.5-1.5)
AUC_{0-23h} (ng×h/mL)		3.54 (37.4%)	3.64 (40.3%)	5.03 (42.0%)	7.33 (41.1%)
GMR		*Control*	1.03 (0.89-1.18)		
GMR			*Control*	1.38** (1.15-1.66)	2.01** (1.79-2.27)
$AUC_{0-\infty}$ (ng×h/mL)		3.75 (35.9%)	3.87 (38.4%)	5.15 (39.8%)	7.65 (36.6%)
GMR		*Control*	1.03 (0.91-1.17)		
GMR			*Control*	1.33** (1.12-1.59)	1.98** (1.79-2.19)
$t_{1/2}$ (h)		1.53 (35.6%)	1.53 (33.2%)	1.46 (26.6%)	1.85 (28.8%)
GMR		*Control*	1.00 (0.84-1.18)		
GMR			*Control*	0.96 (0.79-1.16)	1.22* (1.06-1.39)

[1] CYP2B6 phenotypes were the following: 1 rapid, 7 normal, 7 intermediate and 1 poor metabolizer, who was excluded from the statistical analyses regarding bupropion and hydroxybupropion.

[2] Calculation of $t_{1/2}$ for hydroxybupropion was not possible for four study participants because there was insufficient number of data points in the elimination phase in at least one study phase.
[3] CYP2C9 phenotypes were the following: 10 normal and 6 intermediate metabolizers.
[4] CYP2C19 phenotypes were the following: 2 ultrarapid, 3 rapid, 5 normal and 6 intermediate metabolizers.
[5] One study participant was excluded due to delayed and erratic absorption of omeprazole in two study phases.
[6] CYP3A4 phenotypes were the following: 14 normal and 2 intermediate metabolizers.
[7] CYP2D6 phenotypes were the following: 1 ultrarapid, 10 normal and 5 intermediate metabolizers.
*P<0.05, **P<0.01.

Table S3. The genotypes, activity scores, and predicted phenotypes of CYP enzymes, *SLCO1B1* (OATP1B1), and *ABCG2* (BCRP) in the 16 study participants. For some genotypes, two alternative genotypes could not be distinguished by the method used and both alternatives are shown.

**Enzyme/transporter**	**Genotype**	**Activity score***	**Phenotype**
CYP2B6	*6/*6 (N=1)		PM
	*1/*6 or *4/*9 (N=6)		IM
	*1/*7 (N=1)		
	*1/*1 (N=6)		NM
	*1/*5 (N=1)		
	*1/*4 (N=1)		RM
CYP2C9	*1/*3 (N=1)	1.0	IM
	*1/*2 (N=5)	1.5	
	*1/*1 (N=10)	2.0	NM
CYP2C19	*2/*17 (N=2)		IM
	*1/*36 or *1/*37 (N=1)		
	*1/*2 (N=3)		
	*1/*1 (N=5)		NM
	*1/*17 (N=3)		RM
	*17/*17 (N=2)		UM
CYP2D6	*2/*5 (N=2)	1.0	IM
	*2/*4 (N=2)	1.0	
	*1/*3 (N=1)	1.0	
	*1/*41 (N=1)	1.25	NM
	*2/*59 (N=1)	1.5	
	*1/*1 (N=4)	2.0	
	*1/*2 (N=3)	2.0	
	*2x2/*9 (N=1)	2.25	
	*1/*2(N3) (N=1)	3.0	UM

CYP3A4	*1/*22 (N=2)	IM
	*1/*1 (N=14)	NM
CYP3A5	*3/*3 (N=12)	PM
	*1/*3 (N=4)	IM
OATP1B1	*15/*15 (N=3)	PF
	*14/*15 (N=1)	DF
	*1/*5 (N=1)	
	*1/*15 or *5/*37 (N=1)	
	*1/*1 (N=6)	NF
	*1/*37 (N=2)	
	*14/*37 (N=1)	
	*1/*20 or *19/*37 (N=1)	
BCRP	c.421AA (N=2)	PF
	c.421CA (N=3)	DF
	c.421CC (N=11)	NF

DF, decreased function, IM, intermediate metabolizer, NF, normal function, NM, normal metabolizer, PF, poor function, PM, poor metabolizer, RM, rapid metabolizer, UM, ultrarapid metabolizer.

*References: Theken, K.N., et al., Clinical Pharmacogenetics Implementation Consortium Guideline (CPIC) for CYP2C9 and Nonsteroidal Anti-Inflammatory Drugs. *Clin Pharmacol Ther.* **108(2)**, 191-200 (2020).

Bell, C.G., et al. Clinical Pharmacogenetics Implementation Consortium (CPIC) guideline for CYP2D6 genotype and use of ondansetron and tropisetron. *Clin Pharmacol Ther.* **102(2)**, 213-218 (2017).

Table S4. AUC ratios and 2-hour and 4-hour metabolic ratios of Geneva cocktail CYP indices (CYP1A2: paraxanthine/caffeine, CYP2B6: OH-bupropion/bupropion, CYP2C9: 4'-OH-flurbiprofen/flurbiprofen, CYP2C19: 5'-OH-omeprazole/omeprazole, CYP2D6: dextrorphan/dextromethorphan, CYP3A4: 1'-OH-midazolam/midazolam and omeprazole sulfone/omeprazole) as geometric mean values (with geometric CV) in different study phases. Geometric mean ratios (GMR) with 90% confidence intervals compared to respective control phase are indicated on the rows below each pharmacokinetic variable.

	**Geneva cocktail**	**Geneva cocktail + repaglinide**	**Clopidogrel pretreatment**	**Gemfibrozil pretreatment**
**CYP1A2 (N=16)**				
2-hour metabolic ratio	0.33 (40.0%)	0.38 (33.2%)	0.36 (48.3%)	0.27 (37.8%)
GMR	*Control*	1.12 (0.95-1.33)		
GMR		*Control*	0.97 (0.79-1.19)	0.72** (0.62-0.85)
4-hour metabolic ratio	0.52 (34.1%)	0.55 (27.1%)	0.54 (41.3%)	0.42 (34.1%)
GMR	*Control*	1.06 (0.92-1.22)		
GMR		*Control*	0.98 (0.82-1.17)	0.76** (0.68-0.86)
AUC_{0-4h} ratio	0.35 (42.1%)	0.39 (31.4%)	0.38 (51.9%)	0.29 (39.9%)
GMR	*Control*	1.14 (0.96-1.36)		
GMR		*Control*	0.97 (0.77-1.21)	0.72** (0.61-0.85)
AUC_{0-12h} ratio	0.54 (30.8%)	0.58 (23.4%)	0.57 (41.4%)	0.45 (34.3%)
GMR	*Control*	1.08 (0.95-1.22)		
GMR		*Control*	0.97 (0.82-1.16)	0.77** (0.69-0.86)
$AUC_{0-\infty}$ ratio	0.92 (38.6%)	0.99 (32.6%)	1.00 (36.3%)	0.91 (27.1%)
GMR	*Control*	1.08 (0.88-1.31)		
GMR		*Control*	1.00 (0.82-1.23)	0.91 (0.74-1.13)
**CYP2B6[1] (N=15)**				
2-hour metabolic ratio	2.16 (38.8%)	2.34 (35.0%)	0.50 (41.5%)	2.38 (44.6%)
GMR	*Control*	1.08 (0.89-1.32)		
GMR		*Control*	0.22** (0.16-0.29)	1.02 (0.78-1.32)

**CYP2B6[1] (N=15)**				
4-hour metabolic ratio	5.26 (53.6%)	5.63 (44.5%)	1.29 (38.0%)	5.97 (43.7%)
GMR	*Control*	1.07 (0.85-1.35)		
GMR		*Control*	0.23** (0.18-0.30)	1.06 (0.83-1.35)
$AUC_{0\text{-}4h}$ ratio	2.54 (45.2%)	2.70 (41.1%)	0.61 (40.8%)	2.86 (43.8%)
GMR	*Control*	1.06 (0.88-1.28)		
GMR		*Control*	0.23** (0.18-0.29)	1.06 (0.85-1.31)
$AUC_{0\text{-}23h}$ ratio	7.46 (41.1%)	7.91 (37.5%)	2.07 (32.0%)	9.52 (39.5%)
GMR	*Control*	1.06 (0.83-1.35)		
GMR		*Control*	0.26** (0.20-0.35)	1.20 (0.91-1.59)
$AUC_{0\text{-}\infty}$ ratio (N=11)[2]	14.7 (42.7%)	14.7 (36.6%)	5.39 (37.7%)	22.4 (35.2%)
GMR	*Control*	1.00 (0.69-1.45)		
GMR		*Control*	0.37** (0.27-0.49)	1.53** (1.26-1.85)
**CYP2C9[3] (N=16)**				
2-hour metabolic ratio	0.046 (26.0%)	0.048 (40.2%)	0.045 (22.6%)	0.078 (25.7%)
GMR	*Control*	1.04 (0.86-1.26)		
GMR		*Control*	0.94 (0.78-1.12)	1.61** (1.32-1.96)
4-hour metabolic ratio	0.053 (24.0%)	0.056 (38.5%)	0.050 (26.2%)	0.088 (53.4%)
GMR	*Control*	1.06 (0.92-1.23)		
GMR		*Control*	0.90 (0.79-1.02)	1.57** (1.22-2.04)
$AUC_{0\text{-}4h}$ ratio	0.044 (24.2%)	0.047 (36.9%)	0.041 (23.9%)	0.070 (30.7%)
GMR	*Control*	1.09 (0.93-1.26)		
GMR		*Control*	0.87 (0.76-0.99)	1.47** (1.23-1.74)
$AUC_{0\text{-}23h}$ ratio	0.040 (28.4%)	0.046 (40.5%)	0.042 (30.5%)	0.073 (29.8%)
GMR	*Control*	1.13 (0.95-1.34)		
GMR		*Control*	0.91 (0.80-1.03)	1.59** (1.32-1.92)

**CYP2C9[3] (N=16)**				
$AUC_{0-\infty}$ ratio	0.044 (23.0%)	0.049 (37.3%)	0.045 (23.8%)	0.071 (27.0%)
GMR	*Control*	1.09 (0.91-1.31)		
GMR		*Control*	0.93 (0.82-1.07)	1.46** (1.25-1.70)
**CYP2C19[4,5] (N=15)**				
2-hour metabolic ratio	1.02 (65.4%)	1.03 (63.9%)	1.01 (56.2%)	1.62 (61.4%)
GMR	*Control*	1.01 (0.79-1.28)		
GMR		*Control*	0.98 (0.77-1.25)	1.57** (1.27-1.93)
4-hour metabolic ratio	2.22 (89.9%)	1.70 (70.1%)	2.01 (83.7%)	3.93 (116%)
GMR	*Control*	0.77 (0.55-1.07)		
GMR		*Control*	1.18 (0.85-1.63)	2.31** (1.51-3.51)
AUC_{0-4h} ratio	0.97 (40.8%)	1.00 (46.3%)	0.90 (42.8%)	1.42 (44.6%)
GMR	*Control*	1.02 (0.91-1.14)		
GMR		*Control*	0.91 (0.83-1.00)	1.43** (1.27-1.61)
AUC_{0-23h} ratio	1.09 (39.4%)	1.15 (44.8%)	1.00 (42.5%)	1.69 (39.8%)
GMR	*Control*	1.05 (0.95-1.17)		
GMR		*Control*	0.87** (0.80-0.94)	1.47** (1.34-1.61)
$AUC_{0-\infty}$ ratio	1.09 (39.3%)	1.15 (44.9%)	0.99 (42.6%)	1.69 (40.0%)
GMR	*Control*	1.05 (0.95-1.16)		
GMR		*Control*	0.87** (0.80-0.94)	1.47** (1.34-1.62)
**CYP2D6[6] (N=16)**				
2-hour metabolic ratio	2.47 (66.7%)	2.33 (68.8%)	2.55 (88.1%)	4.12 (80.8%)
GMR	*Control*	0.95 (0.76-1.18)		
GMR		*Control*	1.09 (0.77-1.54)	1.77** (1.32-2.36)
4-hour metabolic ratio	1.86 (75.3%)	1.71 (76.8%)	1.75 (90.8%)	3.25 (92.1%)
GMR	*Control*	0.92 (0.75-1.14)		
GMR		*Control*	1.02 (0.76-1.37)	1.90** (1.38-2.61)

**CYP2D6[6] (N=16)**				
$AUC_{0\text{-}4h}$ ratio	2.42 (70.2%)	2.30 (78.5%)	2.46 (96.4%)	4.13 (84.0%)
GMR	*Control*	0.95 (0.78-1.15)		
GMR		*Control*	1.07 (0.78-1.47)	1.79** (1.33-2.41)
$AUC_{0\text{-}23h}$ ratio	1.65 (94.0%)	1.64 (105%)	1.61 (113%)	2.93 (109%)
GMR	*Control*	1.00 (0.80-1.24)		
GMR		*Control*	0.98 (0.70-1.38)	1.79** (1.23-2.59)
$AUC_{0\text{-}\infty}$ ratio	1.50 (88.6%)	1.43 (87.2%)	1.50 (110%)	2.62 (107%)
GMR	*Control*	0.96 (0.75-1.23)		
GMR		*Control*	1.05 (0.76-1.45)	1.83** (1.34-2.48)
**CYP3A4[7] (Midazolam, N=16)**				
2-hour metabolic ratio	0.48 (35.4%)	0.53 (41.9%)	0.53 (46.9%)	1.10 (33.9%)
GMR	*Control*	1.10 (0.94-1.28)		
GMR		*Control*	1.00 (0.87-1.15)	2.08** (1.85-2.35)
4-hour metabolic ratio (N=14)[8]	0.45 (34.2%)	0.53 (41.4%)	0.53 (46.8%)	1.24 (64.2%)
GMR	*Control*	1.18 (0.95-1.48)		
GMR		*Control*	0.99 (0.85-1.15)	2.33** (1.87-2.90)
$AUC_{0\text{-}4h}$ ratio	0.41 (32.5%)	0.48 (38.2%)	0.51 (40.5%)	0.94 (35.4%)
GMR	*Control*	1.19* (1.04-1.35)		
GMR		*Control*	1.04 (0.93-1.18)	1.95** (1.76-2.15)
$AUC_{0\text{-}23h}$ ratio	0.36 (37.0%)	0.43 (38.2%)	0.45 (47.0%)	0.91 (42.6%)
GMR	*Control*	1.20* (1.04-1.39)		
GMR		*Control*	1.03 (0.88-1.20)	2.11** (1.87-2.38)
$AUC_{0\text{-}\infty}$ ratio	0.38 (36.6%)	0.46 (39.0%)	0.46 (45.8%)	0.96 (38.9%)
GMR	*Control*	1.21* (1.06-1.38)		
GMR		*Control*	1.00 (0.86-1.15)	2.08** (1.89-2.30)

**CYP3A4[5,7] (Omeprazole, N=15)**				
2-hour metabolic ratio	0.40 (69.4%)	0.43 (62.4%)	0.53 (71.7%)	0.71 (64.4%)
GMR	*Control*	1.08 (0.68-1.73)		
GMR		*Control*	1.23 (0.77-1.96)	1.65** (1.18-2.29)
4-hour metabolic ratio	1.61 (103%)	1.14 (69.8%)	1.88 (105%)	2.01 (129%)
GMR	*Control*	0.71 (0.41-1.23)		
GMR		*Control*	1.64 (0.89-3.01)	1.76 (1.06-2.92)
AUC_{0-4h} ratio	0.42 (36.6%)	0.46 (41.6%)	0.49 (39.9%)	0.61 (41.0%)
GMR	*Control*	1.09 (0.81-1.46)		
GMR		*Control*	1.06 (0.83-1.35)	1.31* (1.08-1.60)
AUC_{0-23h} ratio	0.63 (37.9%)	0.70 (30.3%)	0.73 (32.9%)	0.83 (33.6%)
GMR	*Control*	1.11 (0.86-1.43)		
GMR		*Control*	1.04 (0.88-1.23)	1.18 (1.02-1.38)
$AUC_{0-\infty}$ ratio	0.63 (38.3%)	0.70 (31.1%)	0.72 (33.2%)	0.82 (34.5%)
GMR	*Control*	1.11 (0.86-1.43)		
GMR		*Control*	1.04 (0.87-1.23)	1.18 (1.01-1.37)

[1] CYP2B6 phenotypes were the following: 1 rapid, 7 normal, 7 intermediate and 1 poor metabolizer, who was excluded from the statistical analyses regarding bupropion and hydroxybupropion.
[2] Calculation of $t_{1/2}$ for hydroxybupropion was not possible for four study participants because there was insufficient number of data points in the elimination phase in at least one study phase.
[3] CYP2C9 phenotypes were the following: 10 normal and 6 intermediate metabolizers.
[4] CYP2C19 phenotypes were the following: 2 ultrarapid, 3 rapid, 5 normal and 6 intermediate metabolizers.
[5] One study participant was excluded due to delayed and erratic absorption of omeprazole in two study phases.
[6] CYP2D6 phenotypes were the following: 1 ultrarapid, 10 normal and 5 intermediate metabolizers.
[7] CYP3A4 phenotypes were the following: 14 normal and 2 intermediate metabolizers.
[8] For two participants 4-hour metabolic ratios could not be calculated as midazolam concentrations were below limit of quantification at the 4-hour sampling point.
*P<0.05, **P<0.01.

Table S5. C_{max} and AUC values of clopidogrel, gemfibrozil and their metabolites as geometric mean values (with 90% confidence intervals).

	**Cmax (ng/mL)**	**AUC[1] (ng×h/mL)**
Clopidogrel	3.75 (2.69-5.24)	11.6 (8.70-15.5)
Clopidogrel carboxylic acid	9 080 (8 520-9 680)	31 200 (28 400-34 200)
Clopidogrel acyl-β-D-glucuronide	2 200 (1 840-2 630)	10 000 (8 340-12 100)
Gemfibrozil	18 200 (15 700-21 100)	56 700 (49 700-64 700)
Gemfibrozil 1-O-β-glucuronide	16 400 (14 600-18 300)	73 700 (64 900-83 900)

[1] AUC_{0-24h} for clopidogrel and its metabolites and AUC_{0-9h} for gemfibrozil and its metabolites.

Table S6. OATP1B1 biomarker GCDCA-3G and OATP1B3 biomarker GCDCA-3S AUC_{0-3h} values (based on concentrations from index drug administration at 9.00 a.m. to sampling time point at 12 a.m. on study days) during repaglinide only, Geneva cocktail only and full cocktail phases. GCDCA-3G parameters were calculated for normal/decreased function OATP1B1 phenotype group (N=13) after exclusion of study participants with poor function OATP1B1 phenotype (N=3). Variables are expressed as geometric mean values (with geometric CV). Geometric mean ratios (GMR) (with 90% confidence intervals) compared to respective control phase are indicated on the rows below each variable. *P*<0.05 is considered statistically significant.

	**Repaglinide**	**Geneva cocktail**	**Geneva cocktail + repaglinide**
**OATP1B1 normal/decreased function (N=13)**			
GCDCA-3G AUC_{0-3h} (ng×h/mL)	68.3 (61.0%)	63.6 (75.2%)	66.3 (49.6%)
GMR	1.03 (0.89-1.19)	0.96 (0.75-1.22)	*Control*
P-value	>0.99	>0.99	
**OATP1B3 (N=16)**			
GCDCA-3S AUC_{0-3h} (ng×h/mL)	119 (69.0%)	106 (65.4%)	141 (68.3%)
GMR	0.84 (0.70-1.00)	0.75 (0.57-1.00)	*Control*
P-value	0.11	0.09	

Table S7. Metabolic ratios at 8.00 a.m. (0-hour metabolic ratio) and AUC_{0-4h} ratios (based on concentrations from pretreatment drug administration at 8.00 a.m. to sampling time point at 12 a.m. on study days) of solanidine and its metabolites M430 and M444 in the phase with three-day gemfibrozil pretreatment compared to control phase. Variables are expressed as geometric mean values (with geometric CV). Geometric mean ratios (GMR) (with 90% confidence intervals) compared to respective control phase are indicated on the rows below each variable. One participant, who did not have detectable solanidine concentrations in the phase with gemfibrozil pretreatment, was excluded from the analysis. $P<0.05$ is considered statistically significant.

	Geneva cocktail + repaglinide	Gemfibrozil pretreatment
**M430/solanidine (N=15)**		
0-hour metabolic ratio	22.0 (139%)	16.2 (128%)
GMR	*Control*	0.74 (0.39-1.40)
P-value		0.42
AUC_{0-4h} ratio	24.7 (111%)	17.1 (135%)
GMR	*Control*	0.69 (0.37-1.28)
P-value		0.31
**M444/solanidine (N=15)**		
0-hour metabolic ratio	41.4 (122%)	33.2 (126%)
GMR	*Control*	0.80 (0.41-1.55)
P-value		0.56
AUC_{0-4h} ratio	42.6 (115%)	33.9 (143%)
GMR	*Control*	0.80 (0.41-1.56)
P-value		0.56
